# Two new species and new provincial records of aleocharine rove beetles from Newfoundland and Labrador, Canada (Coleoptera, Staphylinidae, Aleocharinae)

**DOI:** 10.3897/zookeys.593.8412

**Published:** 2016-05-26

**Authors:** Jan Klimaszewski, David W. Langor, Caroline Bourdon, Amélie Gilbert, Myriam Labrecque

**Affiliations:** 1 Natural Resources Canada, Canadian Forest Service, Laurentian Forestry Centre, 1055 du P.E.P.S., P.O. Box 10380, Stn. Sainte-Foy, Québec, Quebec, Canada G1V 4C7; 2 Natural Resources Canada, Canadian Forest Service, Northern Forestry Centre, 5320-122 Street, Edmonton, Alberta, Canada T6H 3S5

**Keywords:** Coleoptera, rove beetles, Staphylinidae, Aleocharinae, new provincial records, new species, Canada, Newfoundland and Labrador

## Abstract

Two new species, *Atheta pseudovestita* Klimaszewski & Langor, **sp. n.**, *Silusa prettyae* Klimaszewski & Langor, **sp. n.**, are described, and 16 new provincial records, including one new country record, of aleocharine beetles are presented for the province of Newfoundland and Labrador. Diagnostics, images of habitus and genital structures, distribution, bionomics information and new locality data are provided for the newly recorded species. A new checklist with 189 species of aleocharines recorded from the province is presented.

## Introduction

In the first comprehensive survey of the Aleocharinae fauna of Newfoundland and Labrador (NL), 172 species in 47 genera and 12 tribes were reported (Klimaszewski et al. 2011). Since this treatment of aleocharine beetles, new specimens have become available for study resulting in the discovery of additional species. Klimaszewski et al. (2015a) published a study of Canadian and Alaskan Clusiota Casey and subgenus Microdota Mulsant & Rey of *Atheta* Thomson with new records of adventive Palaearctic Atheta (Microdota) subtils Scriba from Labrador and New Brunswick. They removed Atheta (Microdota) pratensis (Mäklin) from the Newfoundland list of species on the basis of misidentification. The present, updated list of aleocharines from Newfoundland stands at 189 (Table 1). In this contribution, two species new to science and 16 new provincial records, including one new country record, are provided. As well, an updated checklist of all species from the province is provided (Table 1).

**Table 1.** Species of Aleocharinae recorded from Newfoundland and Labrador and their provincial distribution within Canada. Provinces in bold denote new records given in the present publication. *Considered adventive in North America. **Distribution status Holarctic.

**TRIBE GYMNUSINI**	
*Gymnusa atra* Casey**	NF, NB, NS, QC, ON, MB, AB, YT, NU, NT, BC. USA: AK
*Gymnusa brevicollis* (Paykull)*	NF
*Gymnusa campbelli* Klimaszewski	NF, NB, QC, ON, MB, SK, YT, NT. USA: AK
*Gymnusa grandiceps* Casey	NF, NB, NS, QC, ON, MB. USA: New England states
*Gymnusa lindrothi* Klimaszewski & Langor	NF
*Gymnusa pseudovariegata* Klimaszewski	NF, NS, NT, BC. USA: AK
*Gymnusa smetanai* Klimaszewski**	NF, ON, MB, NT, YT. USA: AK
**TRIBE DEINOPSINI**	
*Deinopsis canadensis* Klimaszewski	NF, ON
*Deinopsis harringtoni* Casey	NF, NB, NS, QC, ON. USA: AK
**TRIBE ALEOCHARINI**	
*Aleochara bilineata* Gyllenhal*	NF, NB, NS, PE, QC, ON, MB, SK, AB, BC. USA: New England states
*Aleochara bimaculata* Gravenhorst	NF, NB, NS, QC, ON, MB, SK, AB, BC. USA: wide distribution
*Aleochara caseyi* Likovský	NF, NB, QC, ON. USA: New England states
*Aleochara castaneipennis* Mannerheim	NF, NB, NS, QC, ON, AB, BC. USA: AK
*Aleochara curtula* (Goeze)*	NF, NB, NS, PE, QC, ON, BC. USA: New England states
*Aleochara fumata* Gravenhorst*	NF, NB, NS, PE, QC, ON, MB, AB, YT, BC. USA: widespread
*Aleochara gracilicornis* Bernhauer	**NL**, NB, NS, QC, ON, MB, SK, AB, YT, NT, BC; USA: AZ, CO, FL, IL, IN, KS, LA, MA, MD, ME, MI, MN, MO, MT, NH, NJ, NM, NY, PA, RI, SD, UT
*Aleochara inexpectata* Klimaszewski **(NPR)**	**NF**, NB, NS, QC, ON. USA: MI, WI
*Aleochara lacertina* Sharp	NF, NB, NS, QC, ON, MB, SK, AB, BC
*Aleochara lanuginosa* Gravenhorst*	NF, NB, NS, QC, ON, AB, BC. USA: AK
*Aleochara litoralis* (Mäklin)	NF, NB, NS, PE, QC, BC. USA: AK
*Aleochara sekanai* Klimaszewski	LB, NB, ON, MB, SK, AB, YT. USA: AK
*Aleochara shelleyae* Klimaszewski & Langor	NF
*Aleochara sculptiventris* (Casey)	NF, NB, QC, ON. USA: widely distributed in the east
*Aleochara tahoensis* Casey **(NPR)**	**NF**, NB, NS, QC, ON, MB, SK, AB, YT, NT, BC
*Aleochara tristis* Gravenhorst*	NF, NB, QC. USA: CA
*Aleochara verna* Say	NF, LB, NB, NS, PE, QC, ON, MB, SK, AB, YT, BC. USA: AK
*Tinotus morion* (Gravenhorst)*	NF, NB, NS, QC, ON, SK, AB, BC. USA: CT, NV
**TRIBE OXYPODINI**	
*Crataraea suturalis* (Mannerheim)*	LB, NB, NS, ON, SK, BC. USA: IL, MA, MO, PA, SC, VA, VT
*Devia prospera* (Erichson)**	LB, NB, ON, SK, AB, YT, NT, BC. USA: AK, CO, MI, MN, NM, OR, SD, UT, WA, WY
*Gnathusa alfacaribou* Klimaszewski & Langor	LB
*Ilyobates bennetti* Donistorphe* (**NPR**)	**NF**, NB, NS, QC
*Meotica pseudowinkleri* Klimaszewski & Langor	NF
*Mniusa minutissima* (Klimaszewski & Langor)	NF, NB
*Neothetalia canadiana* Klimaszewski	NF, QC, YT, BC. USA: AK
*Ocyusa canadensis* Lohse	NF, NB, ON, SK, YT. USA: AK
*Oxypoda brachyptera* Stephens*	NF, NB, NS, QC, ON
*Oxypoda canadensis* Klimaszewski	NF, QC, ON, MB, AB, NT
*Oxypoda convergens* Casey	NF, NB, NS, QC, ON, AB. USA: IA, MO, NY
*Oxypoda demissa* Casey	NF, NS, QC, ON
*Oxypoda frigida* Bernhauer	NF, NB, NS, QC, ON, YT, NT, BC. USA: AK
*Oxypoda grandipennis* (Casey)	NF, LB, NB, NS, QC, ON, SK, AB, YT, BC. USA: AK, NH
*Oxypoda hiemalis* Casey	NF, LB, NB, NS, QC, ON, AB, NT. USA: AK
*Oxypoda inimica* Casey	NF, NB, QC, NT. USA: MA
*Oxypoda lacustris* Casey	NF, LB, NB, NS, QC, ON, MB, SK, AB, YT, NT, BC. USA: AK
*Oxypoda lucidula* Casey	NF, QC, ON, MB, AB, YT, NT. USA: AK, IA, MO, NH, NY
*Oxypoda opaca* (Gravenhorst)*	NF, NS, ON, BC. USA: NC, NY, SC, VT
*Oxypoda operta* Sjöberg*	NF, NS, QC, ON, AB. USA: NH
*Oxypoda orbicollis* Casey	LB, NB, NS, QC, ON, SK, AB, YT. USA: WI
*Oxypoda pseudolacustris* Klimaszewski	NF, NB, NS, QC, ON, SK, AB
*Parocyusa americana* (Casey) **(NPR)**	**NF**, ON. USA: NY
*Parocyusa fuliginosa* (Casey)	NF. USA: NC
*Phloeopora canadensis* Klimaszewski & Langor	NF
**TRIBE TACHYUSINI**	
*Brachyusa helenae* (Casey)	NF, NT. USA: AK
*Gnypeta atrolucens* Casey	NF, LB, QC. USA: NY
*Gnypeta caerulea* (C.R. Sahlberg)**	NF, LB, NB, NS, PE, QC, ON, MB, SK, AB, YT, BC. USA: AK
*Gnypeta carbonaria* (Mannerheim)**	NF, NB, QC, ON, MB, SK, AB, NT. USA: AK
*Gnypeta minuta* Klimaszewski & Webster	NF, NB
*Gnypeta nigrella* (LeConte)	NF, NB. USA: MA, PA, MD, VT
*Gnypeta selmani* Brundin**	NF, LB, QC, MB, SK, YT, NT. USA: AK
*Tachyusa americanoides* Paśnik	NF, NB, ON, MB, AB, NT, BC. USA: NH, NY, MA
**TRIBE BOREOCYPHINI**	
*Boreocypha websteri* Klimaszewski & Langor	NF, LB, NB
**TRIBE MYLLAENINI**	
*Myllaena arcana* Casey	NF, LB, NB, NS, QC, ON, SK, AB. USA: AL, FL, IA, IL, MA, NH, NJ. Mexico.
*Myllaena audax* Casey	NF, NB, QC, ON, NT, BC. USA: IL, LA, MA, NJ, NY, OR, RI, UT, WA
*Myllaena insomnis* Casey	NF, LB, NB, NS, QC, ON, MB, SK, AB, YT, BC. USA: AK, ID, MA, MN, WI
*Myllaena procidua* Casey **(NPR)**	**NF**, NB, QC. USA: MA, MD, VA
**TRIBE AUTALIINI**	
*Autalia rivularis* (Gravenhorst)*	NF, NB, NS, QC, ON, AB, BC. USA: NH
**TRIBE HOMALOTINI**	
*Gyrophaena affinis* Mannerheim*	NF, NB, NS, QC, MB, BC. USA: DC, IA, IL, IN, KY, MA, ME, MI, MN, MO, NC, NH, NJ, NM, NY, PA, TN, WA, WI, WV
*Gyrophaena antennalis* Casey	NF, NB, NS. USA: MA, NC, NY
*Gyrophaena chippewa* Seevers	NF, NB. USA: MI, NC, WI
*Gyrophaena criddlei* Casey	LB, NB, ON, MB, SK, YT
*Gyrophaena insolens* Casey	NF, LB, NB, ON, MB, SK, BC. USA: MI
*Gyrophaena involuta* Casey	NF, NB. USA: MA, ME, NY, WI
*Gyrophaena keeni* Casey	NF, NB, QC, ON, AB, YT, BC. USA: FL, MA, MT, NH, NY, TN, WA, WI
*Gyrophaena laetula* Casey	NF, NB. USA: DC, IL, IN, KY, MA, NY, PA, TN, VA, WI
*Gyrophaena modesta* Casey	NF, NB, NS. USA: IL, IN, MI, MN, NH
*Gyrophaena nana* (Paykull)**	NF, ON, MB, YT, BC. USA: AK, MA, ME, MI, MT, WI, WY
*Gyrophaena nanoides* Seevers	NF, NB, QC. USA: MI, NC, NY, PA
*Gyrophaena neonana* Seevers	NF, YT. USA: NC, PA, WI
*Homalota plana* (Gyllenhal)*	NF, NB, NS, AB. USA: AK
*Leptusa brevicollis* Casey	NF, NB, NS, PE, QC, ON. USA: MA, NC, NH, NY, PA, VA, VT
*Leptusa canonica* Casey	NF, NS, QC, ON. USA: IA, MS, OH, PA, TX
*Leptusa gatineauensis* Klimaszewski & Pelletier	NF, NS, QC, ON, BC
*Leptusa opaca* Casey	NF, NB, NS, PE, QC, ON. USA: AR, GA, NC, NY, PA, RI, WI
*Silusa californica* Bernhauer	NF, NB, NS, QC, AB, BC. USA: AK, CA, MN
*Silusa densa* Fenyes	NF, AB. USA: CA
*Silusa prettyae* Klimaszewski & Langor, **sp. n. (NCR, NPR)**	**NF**
*Silusida marginella* (Casey)	NF, NB, NS, ON. USA: CA, IA, NY, PA
**TRIBE PLACUSINI**	
*Placusa incompleta* Sjöberg*	NF, NB, NS, QC, ON, AB, BC. USA: WA
*Placusa tacomae* Casey	NF, NB, NS, QC, ON, AB, YT, NT, BC. USA: AZ, MA, WA, WI
**TRIBE ATHETINI**	
*Acrotona sequestralis* Klimaszewski & Langor	NF. USA: IA
*Acrotona pseudopygmaea* Klimaszewski & Langor	NF
*Alevonota gracilenta* (Erichson) **(NPR)**	**NF**, NB, ON
*Aloconota sulcifrons* (Stephens)*	NF, NB, QC, ON. USA: AL, IL, IN, KY, MO, NH, NY, TN, VA, WV
*Aloconota neocambrica* Klimaszewski & Langor	NF, LB, NB
*Amischa analis* (Gravenhorst)*	NF, NB, NS, PE, ON. USA: CA, IN, PA
*Atheta acadiensis* Klimaszewski & Majka	NF, NB, NS, PE, QC
*Atheta altaica* Bernhauer**	NF, YT, NT. USA: AK
*Atheta amicula* (Stephens)*	NF, NS. USA: WA
*Atheta annexa* Casey	NF, NB, NS, QC, ON. USA: AL, FL, GA, IA, IL, IN, KS, KY, LA, MO, MS, NC, NY, OH, TN, VA, WI, WY
*Atheta atramentaria* (Gyllenhal)*	NF
*Atheta avalon* Klimaszewski & Langor	NF
*Atheta borealis* Klimaszewski & Langor	NF
*Atheta burwelli* (Lohse)	NF, NB, QC
*Atheta campbelli* (Lohse)	NF, YT. USA: AK
*Atheta capsularis* Klimaszewski	NF, NB, QC
*Atheta caribou* (Lohse)	NF, YT
*Atheta celata* (Erichson)**	NF, NB, NS, QC, SK, BC. USA: AK
*Atheta circulicollis* Lohse	NF, QC
*Atheta crenuliventris* Bernhauer [=*bradorensis* (Lohse)]	NF, NB, QC. USA: ME
*Atheta cryptica* (Lohse)	NF, QC, YT, BC
*Atheta curvipennis* Klimaszewski & Langor	NF, LB
*Atheta dadopora* Thomson**	NF, LB, NB, NS, PE, ON, SK, AB, YT, BC. USA: AK, NY, PA, RI
*Atheta districta* Casey	NF, NB, NS, BC
*Atheta fanatica* Casey	NF, NB, NS, QC, BC. USA: AK, NV
*Atheta frosti* Bernhauer	NF, NB, NS, QC, ON, BC. USA: MA, NC, NH, NY, PA, RI, VT
*Atheta giguereae* Klimaszewski & Webster **(NPR)**	**NF**, NB, NS, ON
*Atheta graminicola* (Gravenhorst)**	NF, NB, QC, ON, MB, AB, YT, NT, BC. USA: AK
*Atheta hampshirensis* Bernhauer	NF, NB, NS, QC, ON, BC. USA: AK, CA, NC, NH, NY, OR, PA, RI, WA
*Atheta klagesi* Bernhauer **(NPR) [redefined**]	**NF**, NB, for the rest of Canada needs to be revised. USA: ME, PA
*Atheta lindrothi* Klimaszewski & Langor	NF
*Atheta longicornis* (Gravenhorst)*	NF, NB, NS, QC. USA: MN
*Atheta nearctica* (Lohse)	NF, YT, NT. USA: AK
*Atheta novascotiae* Klimaszewski & Majka	NF, NB, NS. Saint-Pierre et Miquelon (France)
*Atheta pecki* Klimaszewski & Langor	LB
*Atheta pennsylvanica* Bernhauer	NF, LB, NB, NS, QC, ON. USA: IN, PA, RI, VA
*Atheta platanoffi* Brundin**	NF, LB, NB, NS, ON, AB, YT, BC. USA: AK
*Atheta prudhoensis* (Lohse)	NF, NB, NS, ON, YT. USA: AK, VT
*Atheta pseudocrenuliventris* Klimaszewski	NF, NB, NS
*Atheta pseudodistricta* Klimaszewski & Langor	NF
*Atheta pseudoklagesi* Klimaszewski & Webster **(NPR) [redefined**]	**NF**, NB, for the rest of Canada needs to be revised.
*Atheta pseudomodesta* Klimaszewski	NF, QC
*Atheta pseudosubtilis* Klimaszewski & Langor	NF, LB, NB, QC, AB, YT
*Atheta pseudovestita* Klimaszewski & Langor, **sp. n. (NCR, NPR)**	**NF**
*Atheta regissalmonis* (Lohse)	NF. USA: AK
*Atheta remulsa* Casey	NF, NB, NS, AB, YT, BC
*Atheta savardae* Klimaszewski & Majka	NF, NB, NS, QC
*Atheta sculptisoma* Klimaszewski & Langor	NF, QC
*Atheta strigosula* Casey	NF, NB, YT. USA: NY
*Atheta subtilis* (Scriba)* **(NPR)**	**LB**, **NB**
*Atheta terranovae* Klimaszewski & Langor	NF, LB, QC
*Atheta ventricosa* Bernhauer	NF, NB, NS, QC, ON, AB, YT, BC. USA: AK, DC, NC, NJ, NY, PA, VT
*Atheta vestita* (Gravenhorst)*	NF, NB, NS
*Boreophilia eremita* (Rey)**	NF, NB. USA: AK
*Boreophilia islandica* (Kraatz)**	NF, AB, NU, YT. USA: AK
*Boreophilia nearctica* Lohse	NF, QC, MB, YT. USA: AK
*Boreophilia ovalis* Klimaszewski & Langor	NF
*Boreostiba frigida* (J. Sahlberg)**	NF, QC, YT, NT. USA: AK
*Boreostiba parvipennis* (Bernhauer)	NF, LB, QC, AB, YT, NT. USA: AK, NH
*Boreostiba websteri* Klimaszewski & Langor	LB, NB
*Callicerus rigidicornis* (Erichson)* **(NPR)**	**NF**, ON
*Clusiota impressicollis* (Bernhauer)	NF, NB, QC, BC
*Dinaraea angustula* (Gyllenhal)*	NF, NB, NS, PE, QC, AB. USA: CA, NY
*Dinaraea pacei* Klimaszewski & Langor	NF, LB, NB, QC, AB, YT, BC. USA: AK
*Dochmonota rudiventris* (Eppelsheim)* or **	NF, NB, YT, NT. USA: ID, MA
*Earota dentata* (Bernhauer)	NF, NB, NS, QC, ON, MB, AB, YT, BC. USA: AK, AL, AZ, CO, IA, IL, NC, NJ, NM, OR, VA, WA
*Geostiba circellaris* (Gravenhorst)*	NF, NB
*Hydrosmecta borealis* Klimaszewski & Langor	NF
*Hydrosmecta newfoundlandica* Klimaszewski & Langor	NF. Miquelon (France)
*Liogluta aloconoides* Lohse	NF, LB, NS, QC, AB, YT
*Liogluta nigropolita* (Bernhauer)	NF, QC, YT
*Liogluta gigantea* Klimaszewski & Langor	LB
*Liogluta intermedia* Klimaszewski & Langor	NF
*Lypoglossa angularis obtusa* (LeConte)	NF, NS, QC. USA: ME, NH
*Lypoglossa franclemonti* Hoebeke	NF, NB, NS, QC, ON, MB, SK, AB, YT. USA: NY, VT
*Mocyta breviuscula* (Mäklin)	NF, NB, NS, MB, YT, NT, BC. USA: AK
*Mocyta fungi* (Gravenhorst)*	NF, NB, NS, PE, QC, ON, YT, BC. USA: AK
*Mocyta luteola* (Erichson) **(NPR)**	**NF**, NB, QC, ON. USA: MA, MN, NY
*Mocyta sphagnorum* Klimaszewski & Webster **(NPR)**	**NF**, NB, QC, ON
*Nehemitropia lividipennis* (Mannerheim)*	NF, NB, NS, PE, QC, ON. USA: CA, LA, MA, MN, NE, NM, NY, PA, VT, TX
*Paragoniusa myrmicae* Maruyama & Klimaszewski	NF, AB, BC
*Philhygra botanicarum* (Muona)**	NF, LB, NB, NS, ON, SK, YT, BC
*Philhygra hygrotopora* (Kraatz)* **(NPR)**	**NF**, NB
*Philhygra jarmilae* Klimaszewski & Langor	NF, NB, ON, SK, YT
*Philhygra larsoni* Klimaszewski & Langor	NF
*Philhygra luridipennis* (Mannerheim)*	NF, NB, ON
*Philhygra malleoides* Lohse	NF, QC, MB, NT. USA: AK
*Philhygra pohli* Klimaszewski &Langor	NF
*Philhygra pseudopolaris* Klimaszewski & Langor	NF, QC, MB, YT, NT. USA: AK
*Philhygra pseudoterminalis* Klimaszewski & Langor	NF
*Philhygra ripicoloides* Lohse	NF, YT, NT
*Philhygra rostrifera* Lohse	LB, SK, NT, YT. USA: AK
*Philhygra sinuipennis* Klimaszewski & Langor	NF, LB, NB, SK, YT
*Philhygra varula* (Casey)	NF, NB, QC, MB, QC
*Seeversiella globicollis* (Bernhauer)	NF, NB, NS, QC, ON, SK, AB, BC. USA: AZ, CO, ID, MN, MT, NH, SD, WI. Mexico. Guatemala
*Stethusa spuriella* (Casey) **(NPR)**	**NF**, ON. USA: DE, GA, FL, IN, NY, OH, PA, MO
*Strigota ambigua* (Erichson)	NF, NS. USA: CA, CO, CT, IA, KS, MO, NC, NJ, NM, NY, TX
*Trichiusa pseudopostica* Klimaszewski & Langor	NF
**TRIBE LOMECHUSINI**	
*Drusilla canaliculata* (Fabricius)*	NF, NB, NS, PE, QC, ON. USA: AK, KY, NY
*Zyras obliquus* (Casey)	NF, NB, NS, QC, ON, MB, AB, BC. USA: MI, MO, NH, NY, OR
**SPECIES REMOVED FROM NF LIST**	
*Atheta pratensis* (Mäklin) [misidentification for *Atheta subtilis*]	USA: AK

## Materials and methods

All specimens in this study were dissected to examine the genital structures. Extracted genital structures were dehydrated in absolute alcohol, mounted in Canada balsam on celluloid micro-slides, and pinned with the specimens from which they originated. Images of the entire body and the genital structures were taken using an image processing system (Nikon SMZ 1500 stereoscopic microscope; Nikon Digital Camera DXM 1200F, and Adobe Photoshop software).

Morphological terminology mainly follows that used by Seevers (1978) and Klimaszewski et al. (2011). The ventral side of the median lobe of the aedeagus is considered to be the side of the bulbus containing the foramen mediale, the entrance of the ductus ejaculatorius, and the adjacent ventral side of the tubus of the median lobe with the internal sac and its structures (this part is referred to as the parameral side in some recent publications); the opposite side is referred to as the dorsal side. In the species descriptions, microsculpture refers to the surface of the upper forebody (head, pronotum and elytra).

### Depository/institutional abbreviations

LFC
 Natural Resources Canada, Canadian Forest Service, Laurentian Forestry Centre, R. Martineau Insectarium, Quebec City, Quebec, Canada 


MUN
 Memorial University of Newfoundland, St. John’s, Newfoundland and Labrador (on long-term loan to D. Langor, Edmonton, Alberta) 


### Abbreviations of Canadian provinces and territories

AB – Alberta


BC – British Columbia


LB – Labrador


MB – Manitoba


NB – New Brunswick


NF – Newfoundland


NS – Nova Scotia


NT – Northwest Territories


NU – Nunavut


ON – Ontario


PE – Prince Edward Island


QC – Quebec


SK – Saskatchewan


YT – Yukon Territory


USA state abbreviations follow those of the US Postal Service.

## Discussion

Of the 189 species currently known from NL, 31 are adventive, 17 Holarctic, and 141 are Nearctic. The high percentage (16.4%) of adventive species is not surprising because NL was one of the first Canadian provinces with well-established trade with Europe dating back to the 17th century. Genera with the highest number of adventive species are *Aleochara* (5 spp.) and *Atheta* (5 spp.), and the tribe Athetini (15 spp., including 5 *Atheta* spp.), which contains the majority of aleocharine species. The relatively high percentage of Holarctic species (8.9%) found in NL is due to the distribution of some Holarctic species at higher latitudes in both North America and Europe (e.g., *Gnypeta*, many *Philhygra*, and some *Atheta*).

Detailed provincial faunal surveys provide a clear and comprehensive biodiversity dataset to establish baseline biodiversity composition where ecosystems are undergoing rapid change due to anthropogenic disturbances and climate change. Species from this family and subfamily are known to be exceptionally good ecological indicators and are increasingly being used to assess ecosystem resistance and resilience in the wake of development and environmental changes (Pohl et al. 2007, 2008, Langor, unpublished data). This paper contributes to improving baseline knowledge of the Aleocharinae in the province of NL.

The extensive sampling efforts for insects in the province to date have resulted in 189 known aleocharine species. Undoubtedly, more will be discovered over time with additional sampling and further taxonomic study. However, we believe that due to intensive sampling efforts in NF and LB conducted in recent years (Langor in Klimaszewski et al. 2011), the vast majority of the most common and widely distributed species are now known, so new future additions to the fauna will likely be species associated with rare or poorly sampled microhabitats. As well, the subarctic and arctic northern part of Labrador is poorly sampled but likely contains yet-unrecorded species from the province.

## New taxonomic records

### ALEOCHARINI Fleming

#### Aleochara (Xenochara) inexpectata

Taxon classificationAnimaliaColeopteraStaphylinidae

Klimaszewski

Figs 1–7

##### Diagnosis.

**Figures 1–7.**
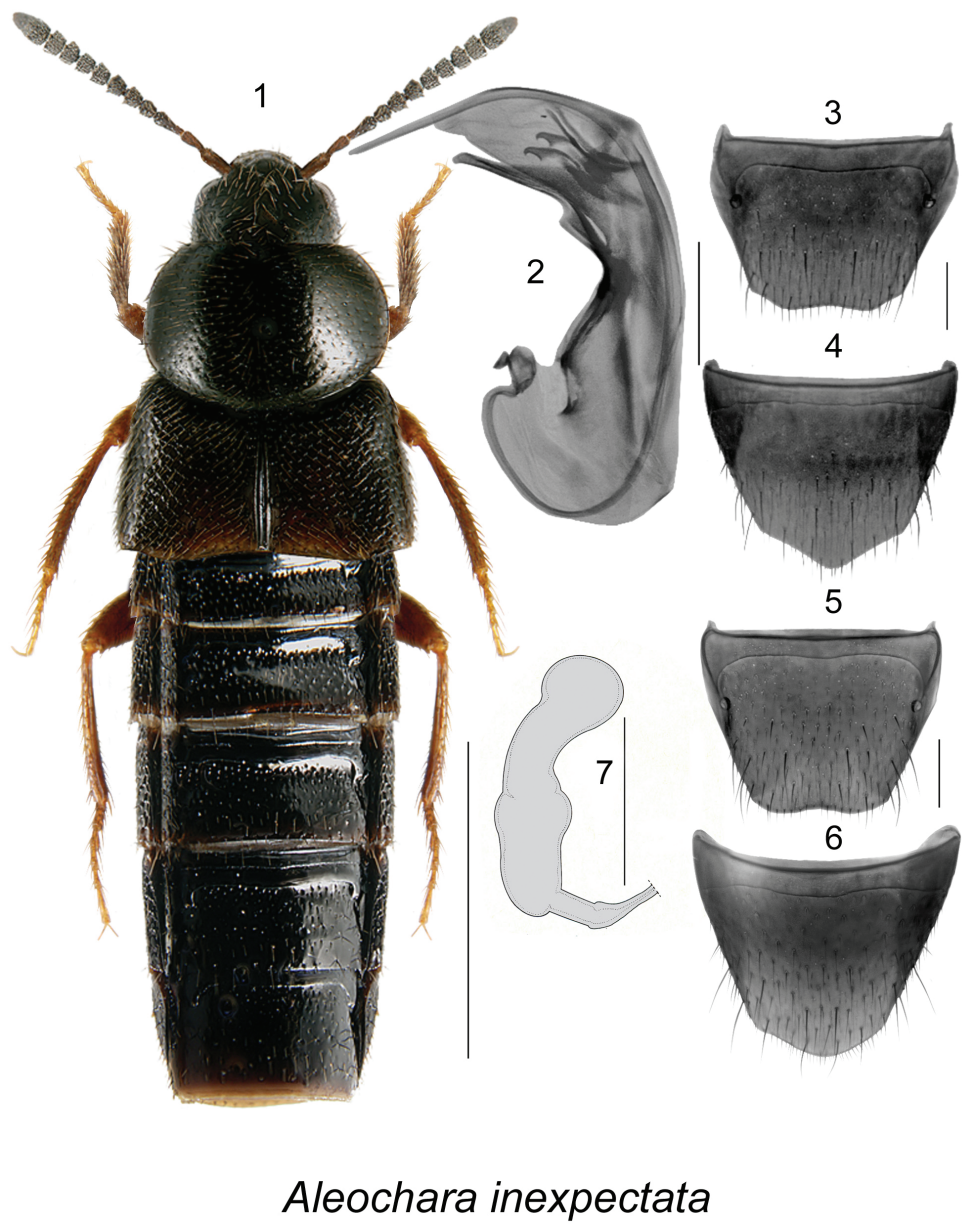
Aleochara (Xenochara) inexpectata Klimaszewski: **1** habitus in dorsal view **2** median lobe of aedeagus in lateral view **3** male tergite VIII **4** male sternite VIII **5** female tergite VIII **6** female sternite VIII **7** spermatheca. Scale bar of habitus = 1 mm; remaining scale bars = 0.2 mm.

Body length 3.0–6.5 mm, piceous-to-black, with tarsi, last articles of labial and maxillary palpi and often posterior margin of elytra rust-brown (Fig. 1). This species is externally very similar to *Aleochara lanuginosa* Gravenhorst from which it differs by the shape of the sclerites of the internal sac of the aedeagus (Fig. 2), the shape of the spermatheca (Fig. 7), and the smooth apical margin of male tergite VIII (Fig. 3). For a more detailed description, see Klimaszewski (1984).

##### Distribution.

**Table:** 

Origin	Nearctic
Distribution	Canada: **NL**, NB, NS, QC, ON; USA: MI, WI
New records	New provincial record; NEWFOUNDLAND: Bog near Burgeo jct., 48.5612°N, 58.2638°W, 26-VI-2011, in moose dung, D. Langor & G. Pohl (MUN) 1 male; Blow Me Down, 49.050°N, 58.251°W, 26-VI-2010, in bear dung, D. Langor (MUN) 3 males; Cape Anguille, 47.899°N, 59.411°W, 22-VI-2010, sheep/horse dung, D. Langor (MUN) 1 male.
References	Klimaszewski 1984, Klimaszewski and Cervenka 1986, Gouix and Klimaszewski 2007, Webster et al. 2009, Brunke et al. 2012, Bousquet et al. 2013

##### Bionomics.

In Newfoundland, adults were collected in moose dung near a bog, and in bear and sheep/horse dung. In New Brunswick, adults were captured from fresh moose dung in an eastern white cedar swamp and in decaying sea wrack resting on vegetation on the upper margin of a salt marsh (Webster et al. 2009). The adults were collected from May to July.

#### Aleochara (Aleochara) tahoensis

Taxon classificationAnimaliaColeopteraStaphylinidae

Casey

Figs 8–14

##### Diagnosis.

**Figures 8–14.**
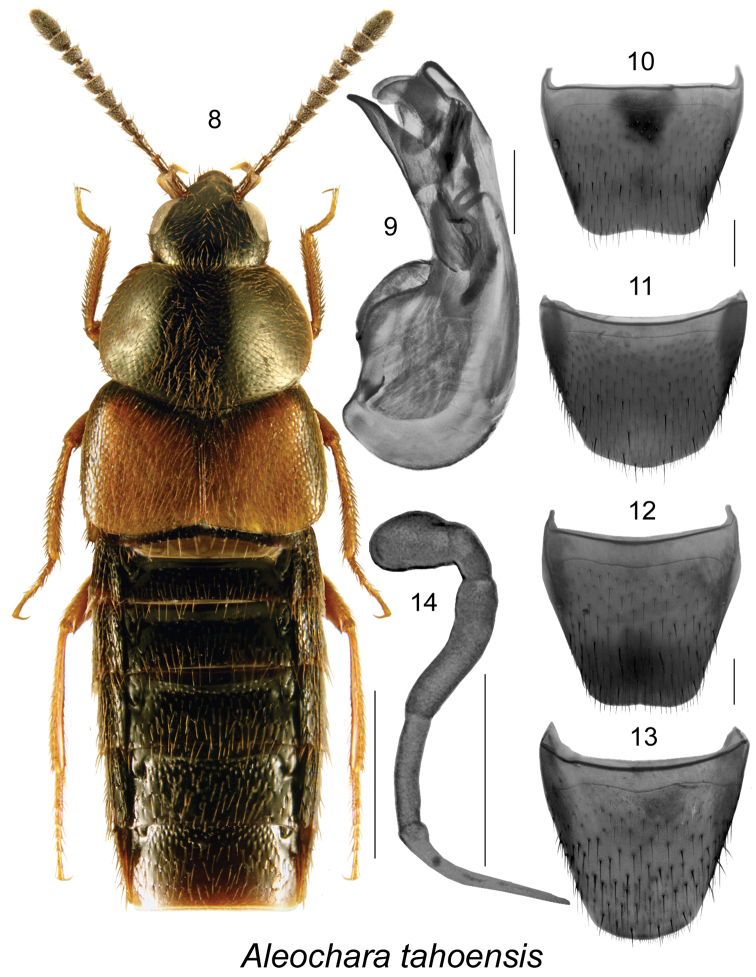
Aleochara (Aleochara) tahoensis Casey: **8** habitus in dorsal view **9** median lobe of aedeagus in lateral view **10** male tergite VIII **11** male sternite VIII **12** female tergite VIII **13** female sternite VIII **14** spermatheca. Scale bar of habitus = 1 mm; remaining scale bars = 0.2 mm.

Body length 4.5–7.0 mm, robust, dark brown to black, with legs, labial and maxillary palpi and most of elytra (except for scutellar section) rust-brown (Fig. 8); maximum distance between eyes equal to 2.5 times maximum diameter of eye (Fig. 8). This species is externally very similar to *Aleochara gracilicornis* Bernhauer from which it differs by having a wider distance between eyes (2.0 times maximum diameter of eye in *Aleochara gracilicornis*). It may be distinguished from all species of *Aleochara* by the shape of median lobe and the sclerites of the internal sac of the aedeagus (Fig. 9), and the shape of the spermatheca (Fig. 14). For a more detailed description, see Klimaszewski (1984).

##### Distribution.

**Table:** 

Origin	Nearctic
Distribution	Canada: **NL**, NB, NS, QC, ON, MB, SK, AB, YT, NT, BC; USA: CA, CO, MO, NH, NM, NV, OR, WA, WI
New records	New provincial record; NEWFOUNDLAND: Terra Nova National Park, Sandy Pond, 54.02°W, 48.49°N, beach rocks and detritus, 14.VIII.2014, D. & M. Langor (MUN) 5 males.
References	Klimaszewski 1984, Gouix and Klimaszewski 2007, Majka and Klimaszewski 2010, Brunke et al. 2012, Bousquet et al. 2013

##### Bionomics.

In Newfoundland, adults were collected from among beach rocks and detritus. Elsewhere, adults were captured from flood debris, swampy areas, debris around dead elm and from a moose carcass (Klimaszewski 1984). Most specimens collected in southwestern USA were found at high altitudes up to 2438 m. The adults were collected from May to September.

#### Aleochara (Aleochara) gracilicornis

Taxon classificationAnimaliaColeopteraStaphylinidae

Bernhauer

Figs 15–22

##### Diagnosis.

**Figures 15–22.**
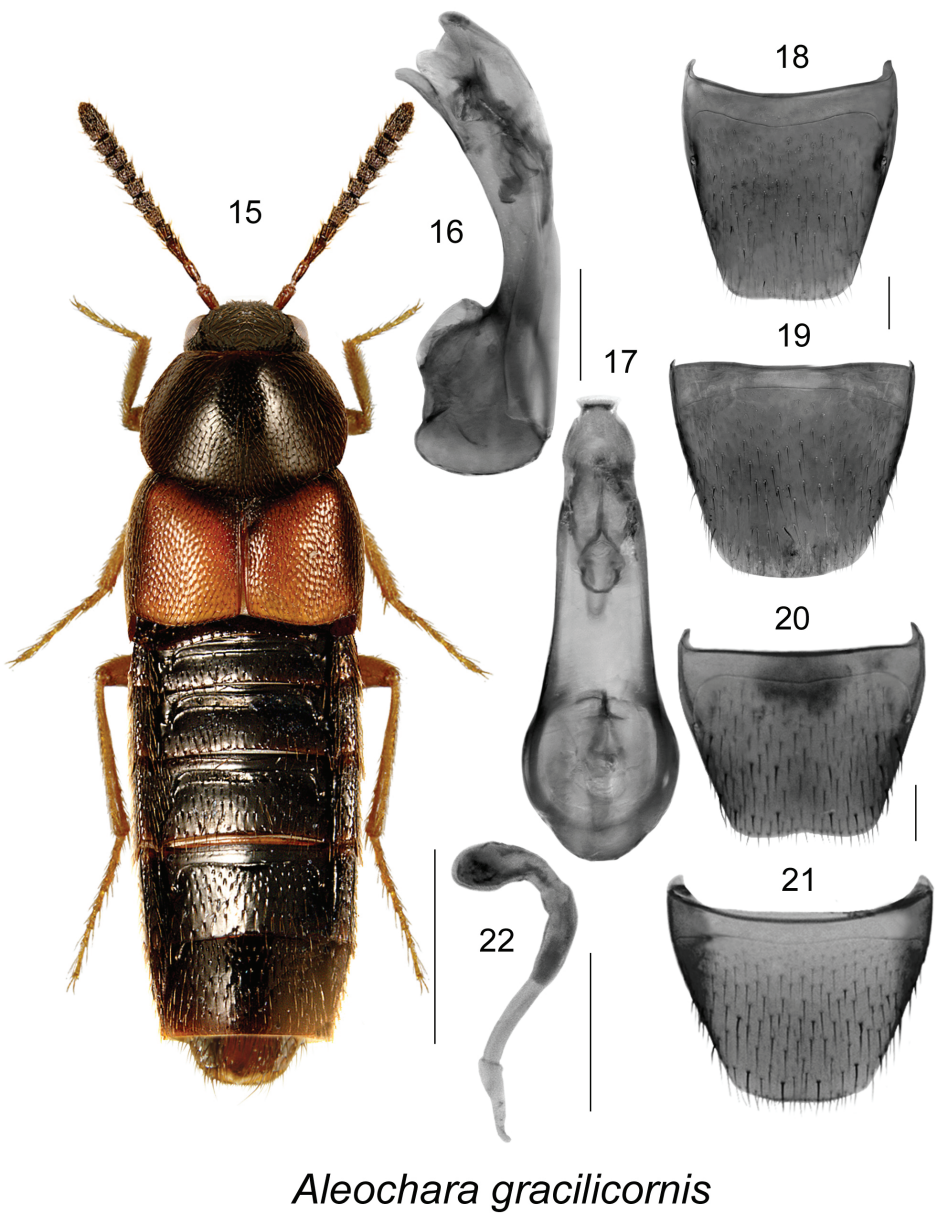
Aleochara (Aleochara) gracilicornis Bernhauer: **15** habitus in dorsal view **16** median lobe of aedeagus in lateral view **17** median lobe of aedeagus in dorsal view **18** male tergite VIII **19** male sternite VIII **20** female tergite VIII **21** female sternite VIII **22** spermatheca. Scale bar of habitus = 1 mm; remaining scale bars = 0.2 mm.

Body length 4.0–6.0 mm, robust, dark brown to black, with legs or only tarsi, labial and maxillary palpi and most of elytra (except sides and for scutellar section) rust-brown to yellowish-brown (Fig. 15); maximum distance between eyes equal to 2.0 times maximum diameter of eye (Fig. 15). This species is externally very similar to *Aleochara tahoensis* Bernhauer from which it differs by having a narrower distance between eyes (2.5 times maximum diameter of eye in *Aleochara tahoensis*). It may be distinguished from all species of *Aleochara* by the shape of the median lobe and the sclerites of the internal sac of the aedeagus (Fig. 16), and the shape of the spermatheca (Fig. 22). For a more detailed description, see Klimaszewski (1984).

##### Distribution.

**Table:** 

Origin	Nearctic
Distribution	Canada: **NL**, NB, NS, QC, ON, MB, SK, AB, YT, NT, BC; USA: AZ, CO, FL, IL, IN, KS, LA, MA, MD, ME, MI, MN, MO, MT, NH, NJ, NM, NY, PA, RI, SD, UT
New records	New provincial record; NEWFOUNDLAND: Badger, N:o 256, 22-25.VI.51, Lindroth (MZH) 1 specimen; Badger, N:o 257, 22-23.VI.51, Lindroth (MZH) 1 specimen; Terra Nova, N:o 327, 26-28.VII.51, Lindroth (MZH) 2 specimens; Millertown, N:o 239, 14.VI.51, Lindroth (MZH) 1 specimen.
References	Klimaszewski 1984, Gouix and Klimaszewski 2007, Bousquet et al. 2013

##### Bionomics.

In North America, adults were collected from debris among vegetation in a temporary creek, from leaves and debris at the edge of deciduous forest and from flood debris, in swampy habitats, and in an old beaver lodge and on carrion (Klimaszewski 1984). Specimens were collected from March to September at altitudes up to 2651 m.

### OXYPODINI Thomson

#### Ilyobates bennetti

Taxon classificationAnimaliaColeopteraStaphylinidae

Donistorphe

Figs 23–30

##### Diagnosis.

**Figures 23–30.**
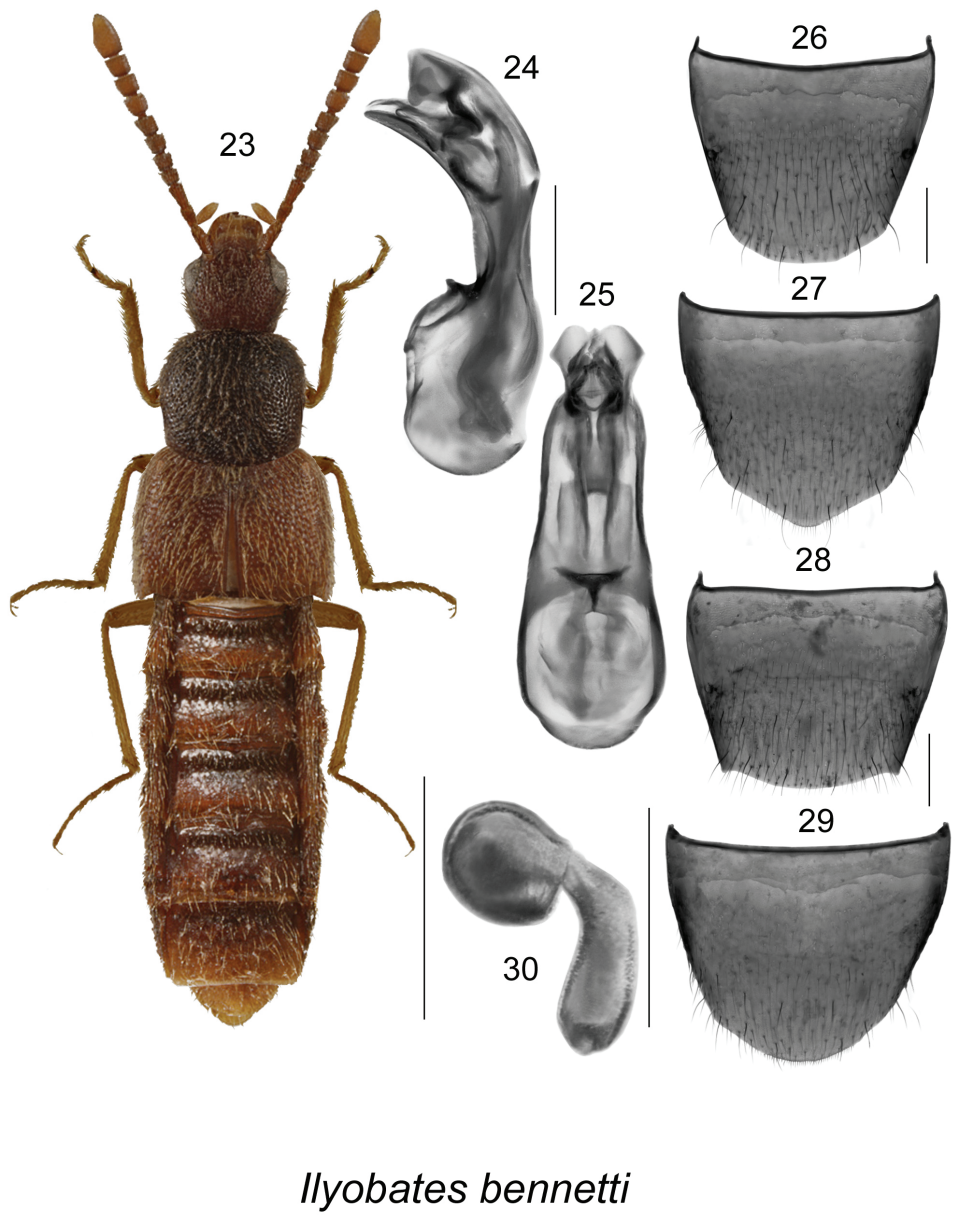
*Ilyobates bennetti* Donistorphe: **23** habitus in dorsal view **24** median lobe of aedeagus in lateral view **25** median lobe of aedeagus in dorsal view **26** male tergite VIII **27** male sternite VIII **28** female tergite VIII **29** female sternite VIII **30** spermatheca. Scale bar of habitus = 1 mm; remaining scale bars = 0.2 mm.

This species is easily distinguishable from other aleocharines by its distinctive body shape, integument with coarse and dense punctation and pubescence (Fig. 23), and the genital structures (Figs 24, 25, 30). Body colour is reddish to almost black.

##### Distribution.

**Table:** 

Origin	Palaearctic, adventive in Canada
Distribution	Canada: **NL**, NB, NS, QC
New records	New provincial record; NEWFOUNDLAND: Barachois Pd. Prov. Pk., 48.483°N, 58.269°W, 11-VII-2011, mixed forest, Heather Beck (MUN) 1 male; Cheeseman Provincial Park, mixedwood boreal forest, 47.633°N, 59.256°W, pitfall trap, 23.VII.2012, Lorna Lafosse (MUN) 3 males, 2 females; same data except: 5.VIII.2012 (MUN) 2 females, 1 sex undetermined.
References	Assing 1999, Majka and Klimaszewski 2008, Webster et al. 2009, Brunke et al. 2012

##### Bionomics.

In Newfoundland, specimens were captured in mixed boreal forest using pitfall traps. In New Brunswick, this adventive species was collected in litter at the base of a tree in a silver maple swamp, in flood debris along a river margin, and among decaying corncobs and cornhusks near a home in a forested residential area (Webster et al. 2009). Majka and Klimaszewski (2008) reported this species from pitfall traps in pastures and a blueberry field in Nova Scotia. In Europe, this species has been reported from similar habitats (Assing 1999). Adults were collected from June to August.

##### Comments.

This adventive species is well established in eastern Canada.

#### Parocyusa americana

Taxon classificationAnimaliaColeopteraStaphylinidae

(Casey)

Figs 31–34

Chilopora americana Casey 1906: 306. As Tetraleucopora: Seevers 1978: 67; Moore and Legner 1975: 493. As Parocyusa: Ashe 2000: 362, Brunke et al. 2012: 197.

##### Diagnosis.

**Figures 31–34.**
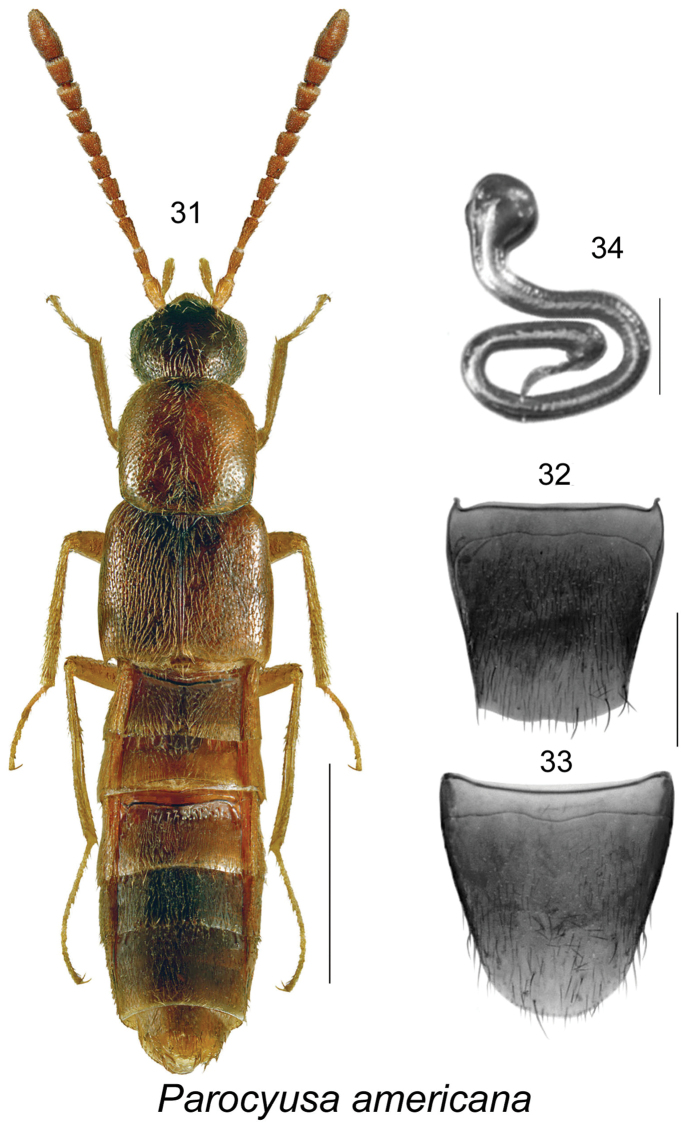
*Parocyusa americana* (Casey): **31** habitus in dorsal view **32** female tergite VIII **33** female sternite VIII **34** spermatheca. Scale bar of habitus = 1 mm; remaining scale bars = 0.2 mm.

This species is easily recognized to genus by the shape of its habitus with subparallel body, deeply impressed and coarsely punctate first three visible abdominal tergites, elongate pronotum, very long tarsi with hind tarsi almost as long as tibia (Fig. 31), and the shape of spermatheca (Figs 34). The only other known Nearctic species, *Parocyusa fuliginosa* (Casey), is darker, with a slightly shorter and more densely punctate pronotum, and has quadrate to slightly transverse antennomeres VIII-X (see Fig. 28 in Klimaszewski et al. 2011).

##### Distribution.

**Table:** 

Origin	Nearctic
Distribution	Canada: **NF**, ON; USA: NY
New records	New provincial record; NEWFOUNDLAND: Glide Lake, 8-IX-1993, pitfall 3.8 (MUN) 1 female.
References	Casey 1906, Moore and Legner 1975, Seevers 1978, Ashe 2000, Brunke et al. 2012

##### Bionomics.

In Newfoundland, one female was captured in a pitfall trap in September from a coniferous forest. In Ontario, females of *Parocyusa americana* were found on a stream bank and in a dry stream bed under a rock (Brunke et al. 2012).

##### Comments.

This is the second record of this species from Canada, and it is much further east than the first record from Ontario by Brunke et al. (2012). We expect *Parocyusa americana* to occur broadly over northeastern North America in riparian habitats. At both Canadian localities only females were captured, and the original description is also based on a female specimen captured in Peekskill, New York (Casey 1906).

### ATHETINI Casey

#### Alevonota gracilenta

Taxon classificationAnimaliaColeopteraStaphylinidae

(Erichson)

Figs 35–43

Homalota gracilenta Erichson 1839:94. As Alevonota: Assing and Wunderle 2008: 172; Brunke et al. 2012: 162; Webster et al. 2016.

##### Diagnosis.

**Figures 35–43.**
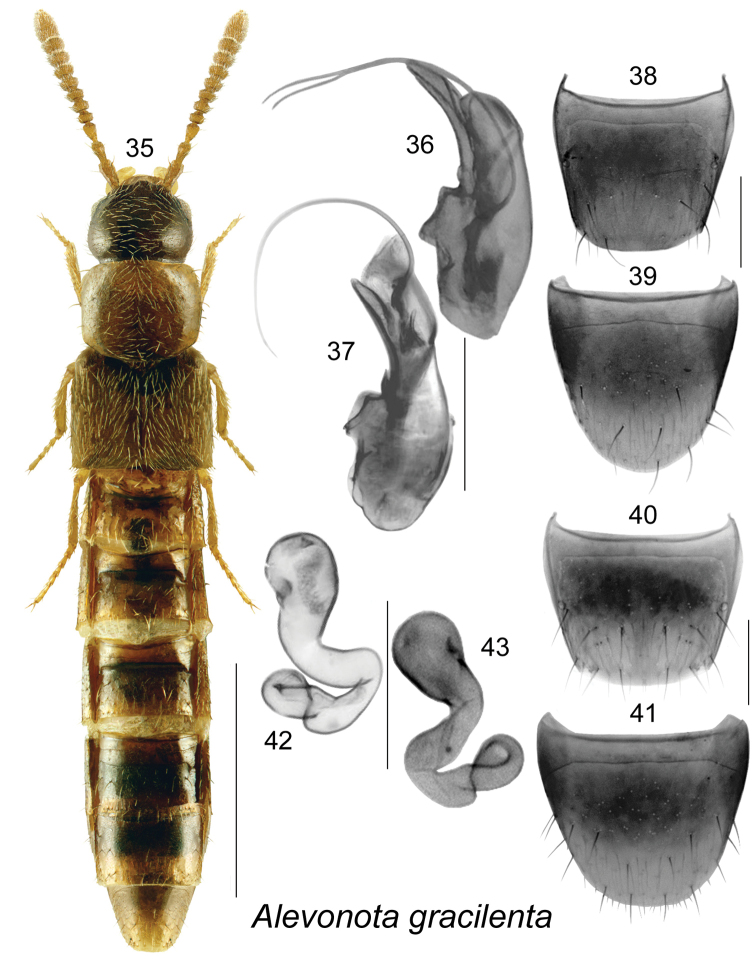
*Alevonota gracilenta* (Erichson): **35** habitus in dorsal view **36, 37** median lobe of aedeagus in lateral view **38** male tergite VIII **39** male sternite VIII **40** female tergite VIII **41** female sternite VIII **42, 43** spermatheca. Scale bar of habitus = 1 mm; remaining scale bars = 0.2 mm.

This species is easily distinguishable from other aleocharines by its small (1.8–3.4 mm) and elongate body (Fig. 35), small eyes, and distinctive genitalia (Figs 36–37, 42–43). Head and abdomen, except for the posterior margins of the segments and the apex, dark brown to blackish; pronotum brown to dark brown; elytra yellowish-brown to brown; legs yellowish; antennae yellowish to yellowish-brown, or rarely the whole body may be considerably darker or paler (Fig. 35). For a more detailed description, see Assing and Wunderle (2008) and Brunke et al. (2012).

##### Distribution.

**Table:** 

Origin	Palaearctic, adventive in Canada
Distribution	Canada: NL, NB, ON
New records	New provincial record; NEWFOUNDLAND: St. John’s, 47.52°N, 52.785°W, Int. Crop 2007/Plot 2, # 191, 7-VII-2007, Peggy Dixon (MUN) 1 male.
References	Erichson 1839, Assing and Wunderle 2008, Brunke et al. 2012, Webster et al. 2016

##### Bionomics.

*Alevonota gracilenta* apparently prefers a wide range of unforested habitats in its native range, but is usually only collected in small numbers and using passive traps (Assing and Wunderle 2008). It was suggested that known specimens represent dispersing individuals and that the real habitat preferences of this species remain unknown, but are possibly subterranean (Assing and Wunderle 2008). In Newfoundland, one male was captured in an agricultural field in July. In New Brunswick, specimens were captured in Lindgren funnel traps in hardwood forests, a mixed forest, and an old white pine (*Pinus strobus* L.) stand. In southern Ontario, specimens were captured in pitfall traps in soybean fields and hedgerows (Brunke et al. 2012). Adults were captured in Canada from May to July.

##### Comments.

The accidental introduction of this obscure Palaearctic species into North America is surprising and may be recent as all known first discovered specimens are from 2009–2010 from two contiguous counties in southern Ontario (Brunke et al. 2012). The presence of this uncommon species in New Brunswick and Newfoundland suggests that it may have been introduced into Canada earlier than previously thought and had been missed due to a lack of adequate sampling in the Atlantic Provinces (Webster et al. 2016). A specimen from Colorado, identified as *Alevonota* by G.A. Lohse, is deposited in the CNC (A. Davies, personal communication) and study of this specimen may reveal that native *Alevonota* species occur in North America (Brunke et al. 2012).

#### Atheta (Dimetrota) giguereae

Taxon classificationAnimaliaColeopteraStaphylinidae

Klimaszewski & Webster

Figs 44–51

##### Diagnosis.

**Figures 44–51.**
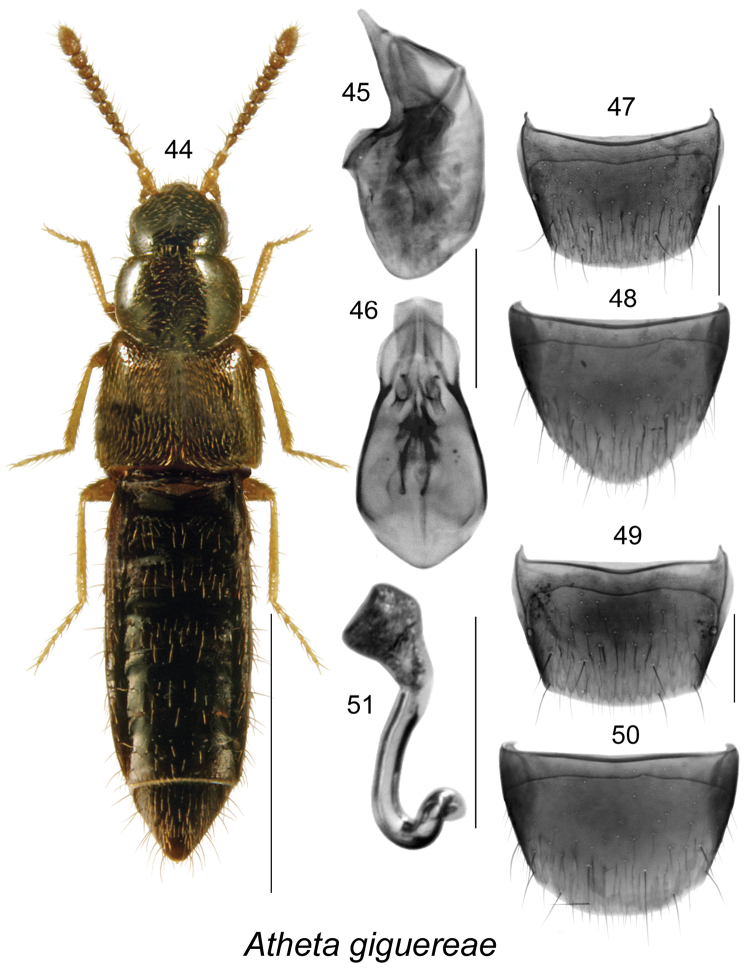
Atheta (Dimetrota) giguereae Klimaszewski & Webster: **44** habitus in dorsal view **45** median lobe of aedeagus in lateral view **46** median lobe of aedeagus in dorsal view **47** male tergite VIII **48** male sternite VIII **49** female tergite VIII **50** female sternite VIII **51** spermatheca. Scale bar of habitus = 1 mm; remaining scale bars = 0.2 mm.

*Atheta giguereae* may be distinguished by the following combination of characters: body length 2.7 mm, narrowly elongate, dark brown with paler legs and basal antennal articles, integument strongly glossy (Fig. 44); median lobe of aedeagus with bulbus narrowly oval, tubus broad, short, and rounded in dorsal view (Fig. 46), and produced ventrally and with apical part triangular in lateral view (Fig. 45); male tergite VIII truncate apically and broadly arcuate (Fig. 47); male sternite VIII almost evenly rounded apically (Fig. 48); female tergite VIII with apical margin arcuate (Fig. 49); sternite VIII broadly rounded apically (Fig. 50); spermatheca with broad pitcher-shaped capsule with large apical invagination and sinuate stem narrowly looped and twisted posteriorly (Fig. 51). For a more detailed description, see Webster et al. (2016).

##### Distribution.

**Table:** 

Origin	Nearctic
Distribution	Canada: **NL**, NB, NS
New records	New provincial record; NEWFOUNDLAND: Cheeseman Provincial Park, 47.633°N, 59.256°W, pitfall trap, 13.VII.2012, Lorna Lafosse (MUN) 1 female.
References	Webster et al. 2016

##### Bionomics.

In Newfoundland, one female was collected in a pitfall trap in a mixed boreal forest in July. In New Brunswick, *Atheta giguereae* was found in mature and old-growth eastern white cedar swamps, a mixed forest, an old-growth northern hardwood forest, and an old white pine stand (Webster et al. 2016). Adults were sifted from moss and leaf litter near streams and brooks and from moist moss in these forests (Webster et al. 2016). A few individuals were captured in Lindgren funnel traps. Specimens from Nova Scotia were captured in flight intercept traps in red spruce and red spruce–hemlock forests (Webster et al. 2016). Adults were collected from April to August.

#### Atheta (Pseudota) klagesi

Taxon classificationAnimaliaColeopteraStaphylinidae

Bernhauer

Figs 52–60

Atheta (s. str.) klagesi Bernhauer 1909: 524. As PseudotaGusarov 2003a: 66; Klimaszewski et al. 2011: 118, Webster et al. 2016.

##### Diagnosis.

**Figures 52–60.**
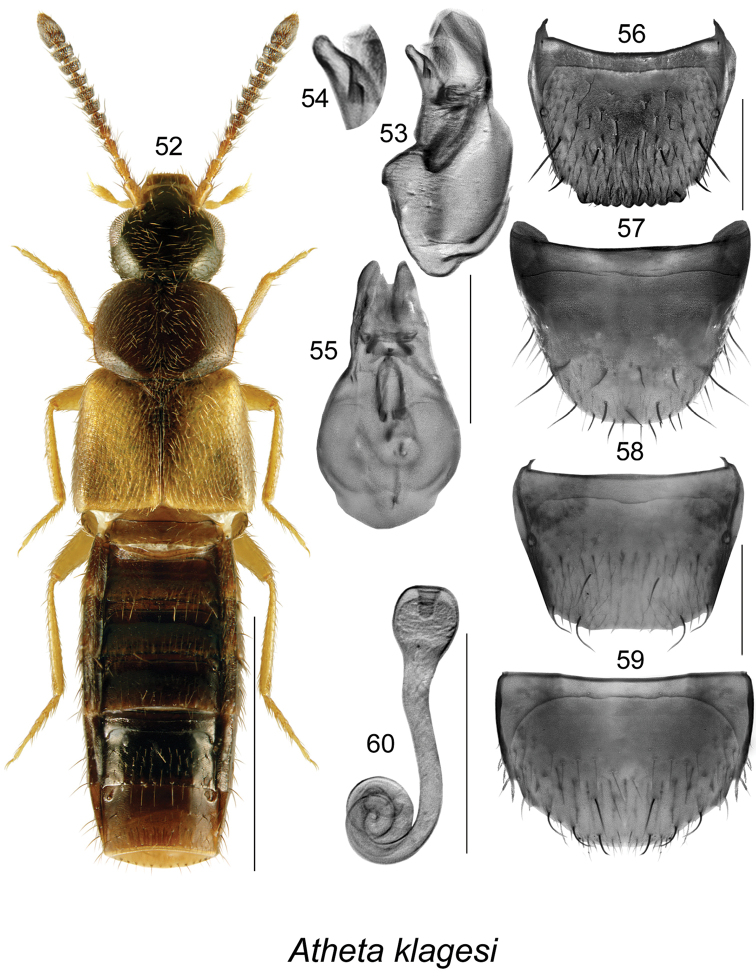
Atheta (Pseudota) klagesi Bernhauer: **52** habitus in dorsal view **53** median lobe of aedeagus in lateral view **54** apical part of tubus of median lobe of aedeagus in lateral view **55** median lobe of aedeagus in dorsal view **56** male tergite VIII **57** male sternite VIII **58** female tergite VIII **59** female sternite VIII **60** spermatheca. Scale bar of habitus = 1 mm; remaining scale bars = 0.2 mm.

*Atheta klagesi* is very similar to *Atheta pseudoklagesi*, and may be distinguished from it by the following combination of characters: body slightly smaller in size and more glossy, yellowish spots on elytra more intense, more intense yellowish colouration of legs, bases of antennae and maxillary palps and overall more contrasting body colour (Fig. 52); median lobe of aedeagus has shorter tubus and a more arcuate and slightly differently shaped apex (Figs 53, 54); spermatheca (Fig. 60) is very similarly shaped in both species and females may be difficult to identify unless collected with males.

##### Distribution.

**Table:** 

Origin	Nearctic
Distribution	Canada: NL, NB; for the rest of Canada and the USA, specimens previously identified as this species need to be re-examined.
Revised records	Revised provincial record; NEWFOUNDLAND: Gallants Rd. 2.2 km from TCH, ARNEWS plot, 48.677°N, 58.195°W, 16-VIII-1995, pitfall trap, W. Bowers (MUN) 2 females; same data as before, except: 31-VII-1995 (MUN) 2 females, 25-VII-1995 (MUN) 3 males; Glide Lake, 15-VIII-1996, trap 1-F-3 (MUN) 1 sex unknown; same data as before, except: trap 3-F-1 (MUN) female; Butterpot Provincial Park, 47.381°N, 53.044°W, pitfall trap, 26.IX.2012, Andrea Pretty (MUN) 1 male.
References	Bernhauer 1909, Gusarov 2003a, Klimaszewski et al. 2011, Webster et al. 2016

##### Bionomics.

In Newfoundland, adults were collected in pitfall traps in boreal conifer forests in July and August.

##### Comments.

See comments under the next species.

#### Atheta (Pseudota) pseudoklagesi

Taxon classificationAnimaliaColeopteraStaphylinidae

Klimaszewski & Webster

Figs 61–68

##### Diagnosis.

**Figures 61–68.**
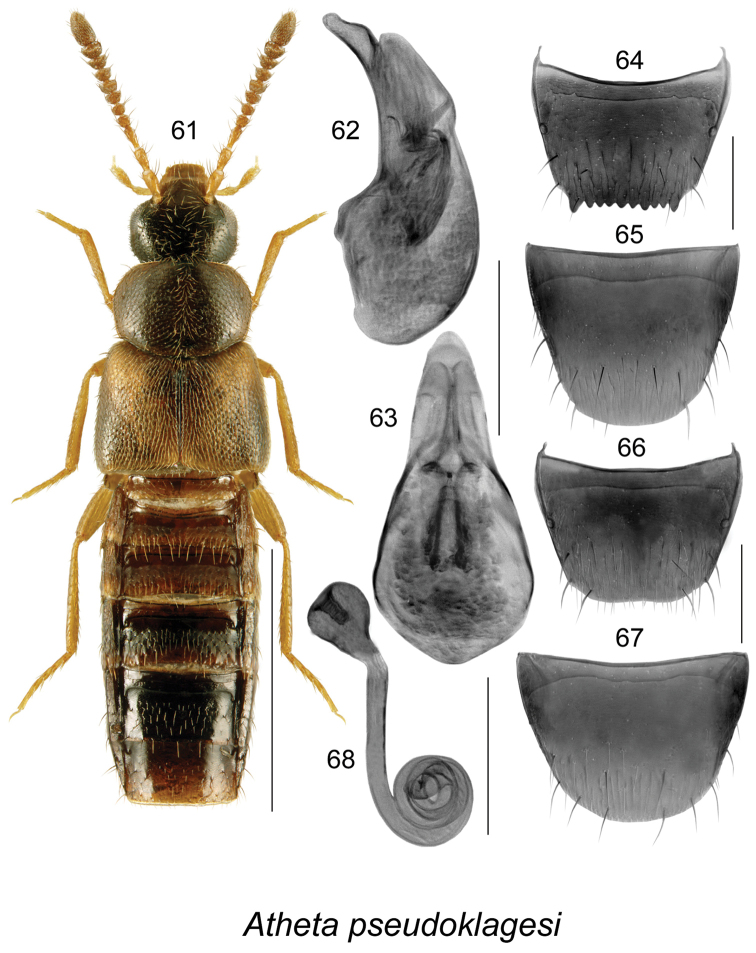
Atheta (Pseudota) pseudoklagesi Klimaszewski & Webster: **61** habitus in dorsal view **62** median lobe of aedeagus in lateral view **63** median lobe of aedeagus in dorsal view **64** male tergite VIII **65** male sternite VIII **66** female tergite VIII **67** female sternite VIII **68** spermatheca. Scale bar of habitus = 1 mm; remaining scale bars = 0.2 mm.

This is a sibling species of *Atheta klagesi* and was previously confused with the latter in collections. It may be distinguished from *Atheta klagesi* by its slightly larger size, less glossy body, less intense yellowish colouration of spots on elytra, legs, bases of antennae and maxillary palps, and overall less contrasting body colour (Fig. 61); median lobe of aedeagus has longer tubus and slightly different shape of apex in lateral view (Fig. 62); spermatheca (Fig. 68) is very similarly shaped in both species and females may be difficult to identify without accompanying males.

##### Distribution.

**Table:** 

Origin	Nearctic
Distribution	Canada: **NL**, NB, for the rest of Canada previously identified specimens must be re-examined.
New records	New provincial record; NEWFOUNDLAND: Gallants, 25-VII-1994, ARNEWS 9-102, W. Bowers (MUN) 1 male; same data as before except: ARNEWS 7-102, 23-VIII-1994 (MUN) 1 male, ARNEWS 9-102, 23-VIII-1994 (MUN) 1 male, ARNEWS 9-102, 26-VII-1994 (MUN) 1 female; Gallants Rd. 2.2 km from TCH, ARNEWS plot, 48.677°N, 58.195°W, 10-VII-1995, pitfall trap, W. Bowers (MUN) 1 male; same data as before except: 25-VII-1995 (MUN) 1 male, 2 female, 23-VIII-1995 (MUN) 3 males, 1 sex unknown, 16-VIII-1995 (MUN) 2 females, 18-VII-1995 (MUN) 1 male; North Harbor, Grand Lake ARNEWS plot, 48.987°N, 57.628°W, 24-VII-1995, pitfall trap, W. Bowers (MUN) 1 female; same data as before, except: 16-VIII-1995 (MUN) 1 female, 23-VIII-1995 (MUN) 1 female, 28-VIII-1995 (MUN) 1 male, 48.988°N, 57.629°W, 10-VII-1995 (MUN) 1 female; Big Bonne Bay Pond ARNEWS Plot, 49.338°N, 57.537°W, 23-VIII-1995, pitfall trap, W. Bowers (MUN) 1 sex unknown.
References	Webster et al. 2016

##### Bionomics.

In Newfoundland, adults were collected in pitfall traps in boreal forests in July and August. In New Brunswick, adults of this species were found in mature mixed forest, old-growth and old white spruce and balsam fir forests, a mature red spruce forest, and in a wet alder swamp. Specimens were collected from coral fungi on a *Populus* log, fleshy polypore fungi at base of a dead standing *Populus*, in decaying gilled mushrooms, in gilled mushrooms, and under bark of red spruce (Webster et al. 2016). Adults were collected from May to September.

##### Comments.

In the past, the two sibling species were mixed together and identified as *Atheta klagesi*. All material across Canada and the USA needs to be re-examined for understanding the true distribution of the two species. In this paper, only Newfoundland and New Brunswick specimens were re-evaluated (Webster et al. 2016).

#### Atheta (Thinobaena) pseudovestita

Taxon classificationAnimaliaColeopteraStaphylinidae

Klimaszewski & Langor sp. n.

http://zoobank.org/26039838-24D6-4BA9-A030-4533193F7EA0

Figs 69–76

##### Holotype

(female). **Canada, Newfoundland**, St. Teresa, 48.3976°N, 58.6201°W, 2 m altitude, 26-VI-2011, under detritus upper beach, D. Langor & G. Pohl (LFC).

##### Paratypes.

**Canada, Newfoundland**: Cape Broyle, 47.0954°N, 52.9525°W, 2 m altitude, 23-VI-2011, in vegetation and gravel on river bank, D. Langor & G. Pohl (MUN) 1 female; Cheeseman Provincial Park, 47.625°N, 59.271°W, 4 m altitude, 23-VI-2011, under beach detritus, D. Langor & G. Pohl (MUN) 1 female; Same data as before except: 47.633°N, 59.255°W, 27-VII-2011, treading marsh shore (LFC) 1 male; same data as before except: 2 m altitude, in detritus along seashore (LFC, MUN) 2 males; Searston, 47.828°N, 59.329°W, 7 m altitude, 23-VI-2011, under seaweed on sandy beach, D. Langor & G. Pohl (MUN) 1 male; Stephenville Crossing, 48.513°N, 58.454°W, 3 m, 22-VI-2011, D. Langor & G. Pohl (LFC, MUN) 2 males, 2 females.

##### Etymology.

*Pseudovestita* is a Latin adjective derived from the specific name of a very similar, adventive Palaearctic species occurring in Newfoundland – *Atheta vestita* (Gravenhorst) and the prefix *pseudo* meaning false.

##### Diagnosis.

**Figures 69–76.**
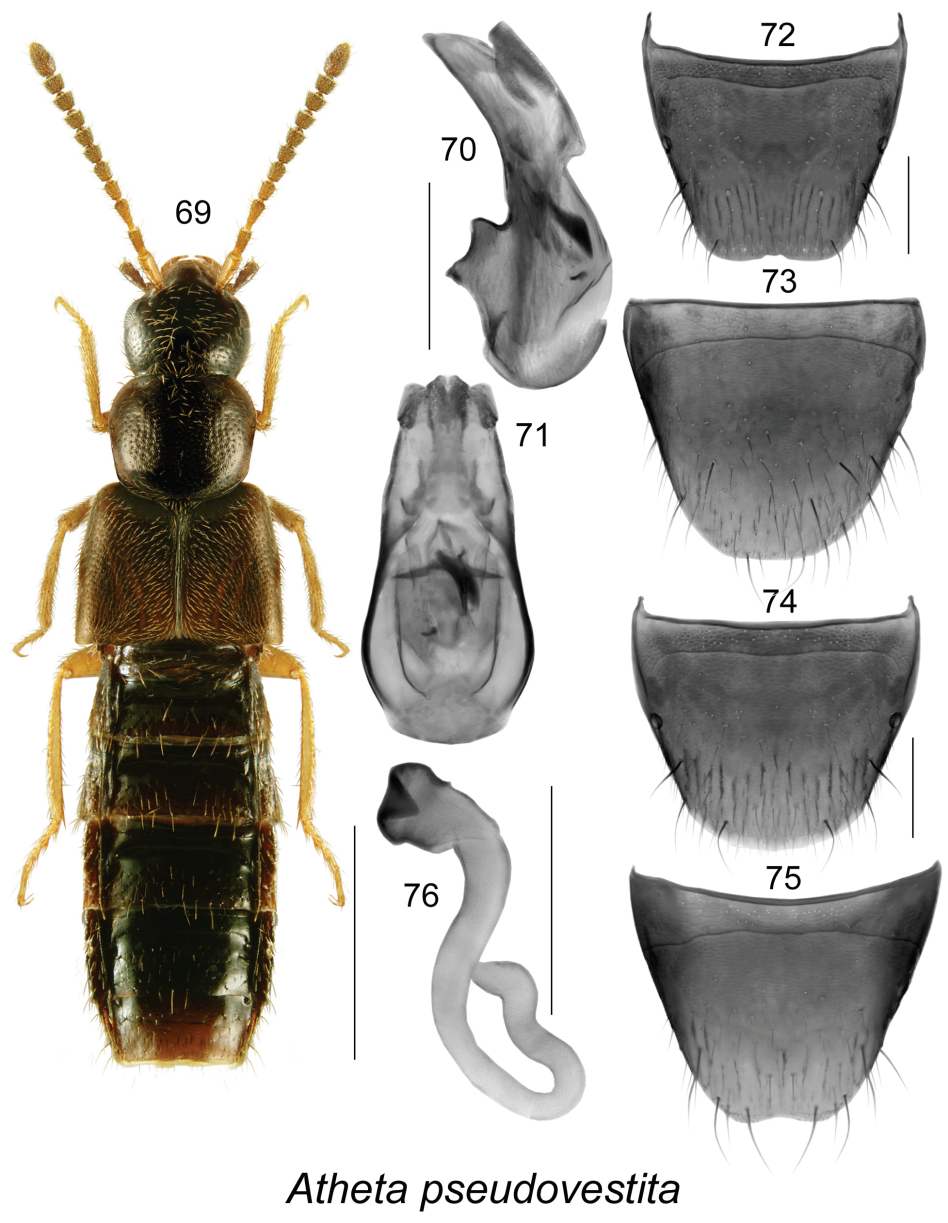
Atheta (Thinobaena) pseudovestita Klimaszewski & Langor, sp. n.: **69** habitus in dorsal view **70** median lobe of aedeagus in lateral view **71** median lobe of aedeagus in dorsal view **72** male tergite VIII **73** male sternite VIII **74** female tergite VIII **75** female sternite VIII **76** spermatheca. Scale bar of habitus = 1 mm; remaining scale bars = 0.2 mm.

Body length 3.5–3.9 mm; body moderately narrow (Fig. 69); head, antennal articles III-XI, pronotum, base of elytra, and abdomen dark brown, but legs and posterior part of elytra paler, yellowish to rust-brown; integument moderately glossy (more so than in *Atheta vestita*), sparsely punctate and pubescent, pubescence short and adhering to the body, with dense meshed microsculpture, denser on forebody, sculpticells hexagonal; head round, about as wide and as long as pronotum, with eyes shorter than postocular area; antennae with articles I-V elongate and VI-X subquadrate to slightly transverse (Fig. 69); pronotum margined laterally, trapezoidal in form in dorsal view, narrowest at base, widening apically to about apical third and then abruptly narrowed apically, slightly transverse, much narrower at base than elytra; elytra flattened, slightly longer than pronotum; abdomen broad, slightly swollen medially. MALE. Median lobe of aedeagus with narrowly oval bulbus streamlined with tubus in dorsal view (Fig. 71); in lateral view tubus arcuate ventrally and with broadly triangular apex (Fig. 70); internal sac structures not pronounced (Figs 70, 71); tergite VIII truncate apically and without teeth (Fig. 72); sternite VIII elongate, broadly rounded apically (Fig. 73). FEMALE. Tergite VIII broadly rounded apically (Fig. 74); sternite VIII truncate apically and slightly emarginated medially (Fig. 75); spermatheca with short capsule bearing wide and relatively deep apical invagination, stem sinuate, bent subapically and sinuate at apex (Fig. 76).

##### Distribution.

Known only from Newfoundland, Canada.

##### Bionomics.

This species was found in Newfoundland under detritus along seashore, under seaweed on sandy beaches, in vegetation and gravel on riverbanks, and on the edge of a marsh very close to a shoreline. Adults were collected in June.

##### Comments.

This species is very similar externally to a Palaearctic adventive species found in NB, NS and NF. Both species may be mixed up in collections. *Atheta pseudovesita* may be distinguished from *Atheta vestita* by the following combination of characters: body distinctly more glossy, colouration darker and predominantly dark brown (light brown in *Atheta vestita*), pubescence on forebody sparser and punctation more distinct, tergites and sternites VIII similar in both species, median lobe of aedeagus narrowly elongate apically in *Atheta vestita* (Fig. 304b in Klimaszewski et al. 2011) and broadly triangular in *Atheta pseudovestita* (Fig. 62); spermatheca of a completely different form, with stem bent and subparallel at 2/3 of its length and with slightly twisted subapical section (Fig. 68), while spermatheca is S-shaped in *Atheta vestita* (Fig. 304c in Klimaszewski et al. 2011). Apparently the two species represent sibling species. For distribution, description and illustrations of *Atheta vestita*, see Klimaszewski et al. 2007, 2011.

#### Callicerus rigidicornis

Taxon classificationAnimaliaColeopteraStaphylinidae

(Erichson)

Figs 77–83

Homalota rigidicornis Erichson 1839: 82. As Callicerus: Assing 2001: 286; Brunke et al. 2012: 175.

##### Diagnosis.

**Figures 77–83.**
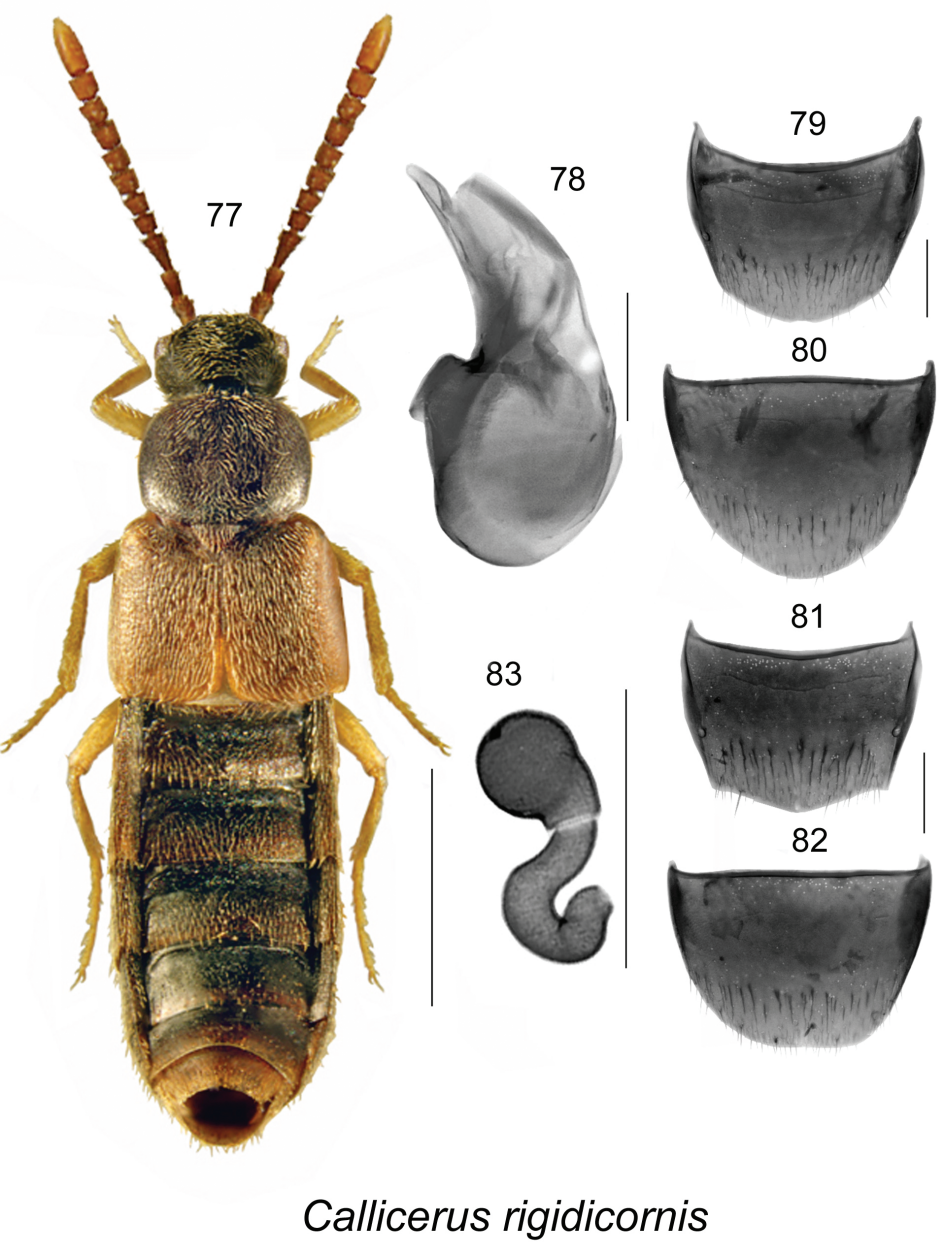
*Callicerus rigidicornis* (Erichson): **77** habitus in dorsal view **78** median lobe of aedeagus in lateral view **79** male tergite VIII **80** male sternite VIII **81** female tergite VIII **82** female sternite VIII **83** spermatheca. Scale bar of habitus = 1 mm; remaining scale bars = 0.2 mm.

There are two adventive species of *Callicerus* reported from Canada (Brunke et al. 2012). Males of *Callicerus rigidicornis* do not have their antennomere X conspicuously elongate (Fig. 77) as in *Callicerus obscurus* (for illustration, see Brunke et al. 2012). *Callicerus rigidicornis* may be distinguished externally from *Callicerus obscurus* by the more transverse pronotum (Fig. 77), larger body (length 3.5–5.0 mm), and by body colouration with lighter basal half of abdomen (entirely dark brown in *Callicerus obscurus*). The habitus and genital structures of *Callicerus rigidicornis* are illustrated in Figs 77–83. For details of European *Callicerus* species, see Assing (2001).

##### Distribution.

**Table:** 

Origin	Palaearctic, adventive in Canada
Distribution	Canada: **NL**, ON.
New records	New provincial record; NEWFOUNDLAND: St. John’s, 47.52°N, 52.785°W, Int. Crop 2007/Plot 1, #187, 2007, Peggy Dixon (MUN), 1 female; Int. Crop 2007/Plot 5, #182, 2007, (MUN), 1 female.
References	Erichson 1839, Assing 2001, Brunke et al. 2012

##### Bionomics.

The Newfoundland females were captured using pitfall traps in agricultural fields in 2007. In Ontario, specimens were collected in agricultural hedgerows using pitfall traps in 2009 and 2010 (Brunke et al. 2012). Adults were collected in May and June.

##### Comments.

*Callicerus rigidicornis* was recorded from North America as an adventive species for the first time based on Ontario specimens collected in agricultural hedgerows (Brunke et al. 2012). The NL record may suggest a broader distribution of this adventive species in Canada, but it is unknown whether these records represent separate introduction events. For information on natural history of this species in Europe, see Assing (2001).

#### Mocyta luteola

Taxon classificationAnimaliaColeopteraStaphylinidae

(Erichson)

Figs 84–92

Homalota luteola Erichson 1839: 114. As Mocyta: Klimaszewski et al. 2015c: 124.

##### Diagnosis.

**Figures 84–92.**
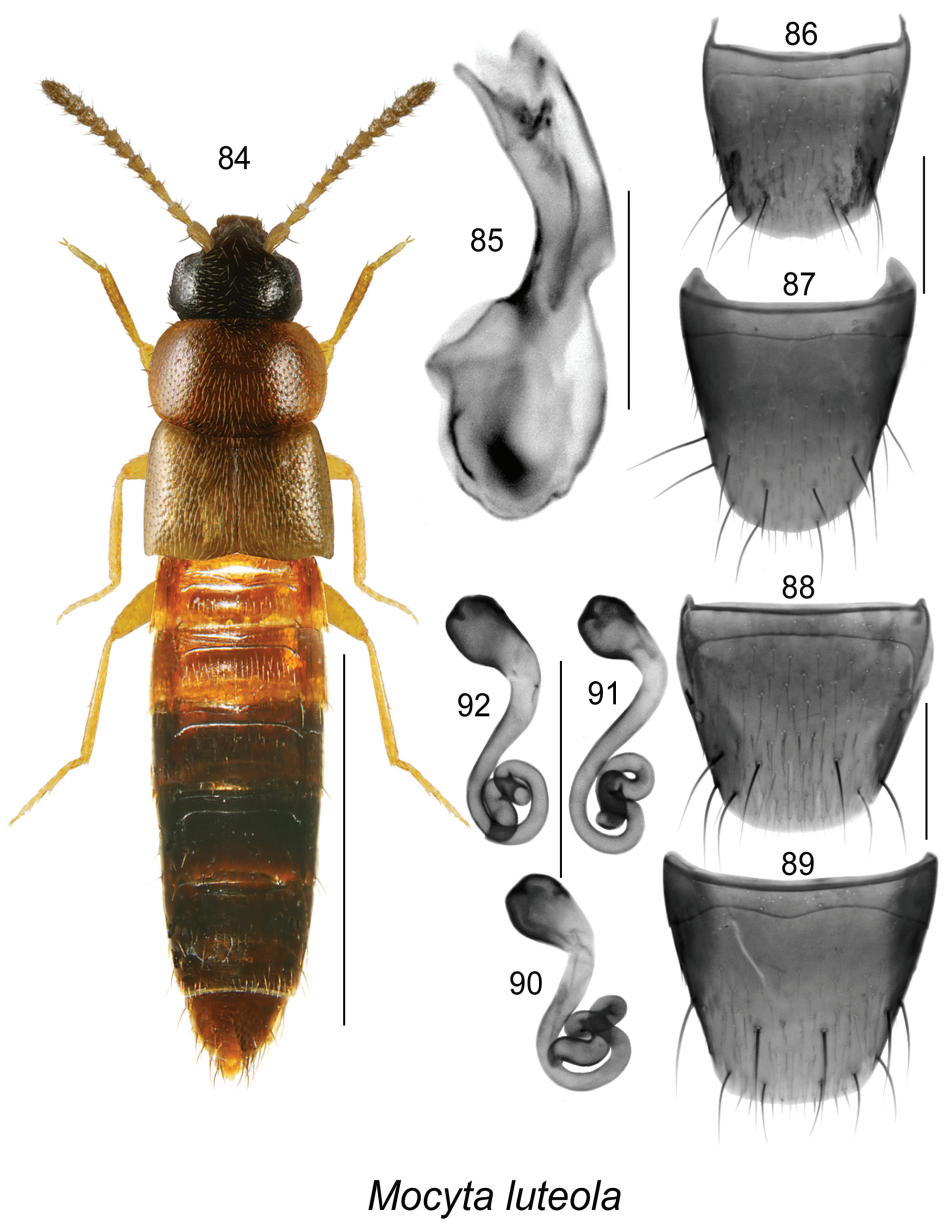
*Mocyta luteola* (Erichson): **84** habitus in dorsal view **85** median lobe of aedeagus in lateral view **86** male tergite VIII **87** male sternite VIII **88** female tergite VIII **89** female sternite VIII **90–92** spermatheca. Scale bar of habitus = 1 mm; remaining scale bars = 0.2 mm.

This species may be distinguishable from other *Mocyta* species by its bicoloured body, dark brown head and posterior part of pronotum contrasting with reddish-brown or yellowish-brown pronotum, elytra, base of abdomen and appendages (Fig. 84), the strong microsculpture of the forebody, and the shape of the median lobe of the aedeagus (Fig. 85). The shape of the spermatheca (Figs 90–92) is similar to that of *Mocyta fungi* (Gravenhorst). For a more detailed description, see Klimaszewski et al. (2015c).

##### Distribution.

**Table:** 

Origin	Nearctic
Distribution	Canada: **NL**, NB, QC, ON. USA: MA, MN, NY
New records	New provincial record; NEWFOUNDLAND: LaManche Prov. Pk., 47.165°N, 52.899°W, 1-VIII-2011, conifer forest, pitfall trap, Doug Harrison (MUN) 1 female.
References	Erichson 1839, Bland 1865, Blatchley 1910, Casey 1910, Moore and Legner 1975, Klimaszewski et al. 2015c

##### Bionomics.

In Newfoundland, one female was captured in a pitfall trap in a boreal conifer forest. Most adults from Quebec were collected in yellow birch- and balsam fir-dominated forest using pitfall traps (Klimaszewski et al. 2007). In New Brunswick, adults were found: under decaying seaweed on a coastal beach; under driftwood on a riverbank; in grass, moss and leaf litter near water and in alder and cedar swamps and *Carex* marshes; in *Sphagnum* moss and leaf litter in a young regenerating mixedwood forest; and in other decaying material in forests. In Ontario, adults were captured in litter around raspberry near a bog, in a *Typha* marsh, and in a nest of *Microtus pennsylvanicus* (Klimaszewski et al. 2015c). Adults were active from March to October in Canada. In Minnesota, adults were captured on a lakeshore and in a *Microtus* nest, and in Indiana were taken by sifting dump vegetable debris from March to November (Blatchley 1910).

##### Comments.

This species is probably more widely distributed in Newfoundland than the single record suggests.

#### Mocyta sphagnorum

Taxon classificationAnimaliaColeopteraStaphylinidae

Klimaszewski & Webster

Figs 93–100

##### Diagnosis.

**Figures 93–100.**
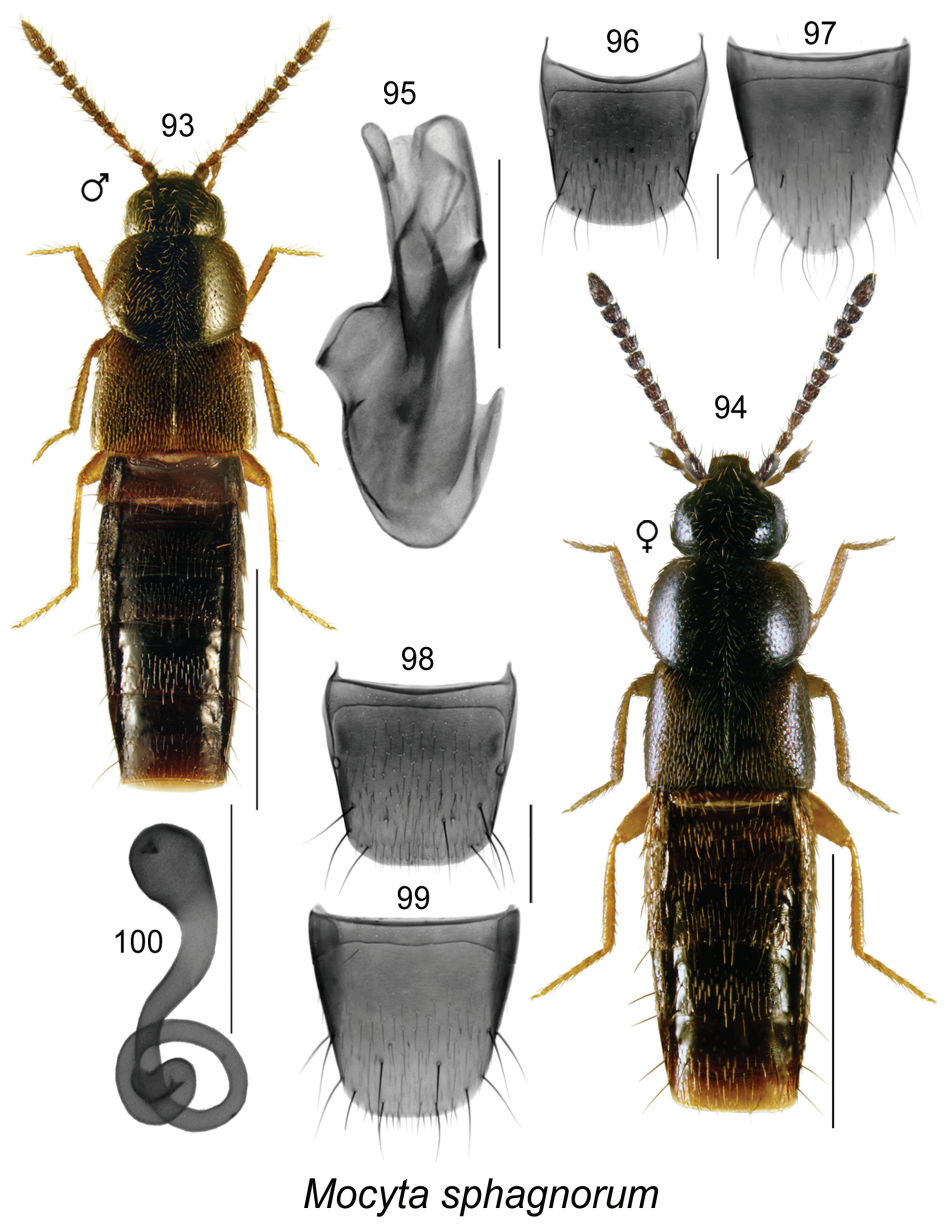
*Mocyta sphagnorum* Klimaszewski & Webster: **93, 94** habitus in dorsal view (male, female) **95** median lobe of aedeagus in lateral view **96** male tergite VIII **97** male sternite VIII **98** female tergite VIII **99** female sternite VIII **100** spermatheca. Scale bar of habitus = 1 mm; remaining scale bars = 0.2 mm.

This species may be distinguishable from other *Mocyta* species by its large and dark brown to black pronotum, elytra about as long as pronotum (Figs 93, 94), shape of apical structures of the internal sac of the aedeagus (Fig. 95), and shape of the spermatheca (Fig. 100). For a more detailed description, see Klimaszewski et al. 2015c.

##### Distribution.

**Table:** 

Origin	Nearctic
Distribution	Canada: **NL**, NB, QC, ON
New records	New provincial record; NEWFOUNDLAND: Gallants Rd. 2.2 km from TCH, ARNEWS plot, 48.677°N, 58.195°W, 28-VIII-1995, pitfall trap, W. Bowers (MUN) 1 female; same data as before, except: 18-VII-1995 (MUN) 1 female, 23-VIII-1995 (MUN) 1 female, 16-VIII-1995 (MUN) 2 females; North Harbor, Grand Lake ARNEWS plot, 48.987°N, 57.628°W, 28-VIII-1995, pitfall trap, W. Bowers (MUN) 1 female; same data as before, except: 18-VII-1995, 1 male; Lockston Path Prov. Pk., 48.430°N, 53.361°W, 18-VII-2011, pitfall trap, P. Perry (MUN) 1 female; Glide Lk, 23-VI-1994, bF cut, trap 4-C-10, Bowers et al. (MUN) 1 male; York Harbour, 49.0555°N, 53.3687°W, 28-VI-2010, under seashore detritus, D. Langor (MUN) 1 female; Cheeseman Provincial Park, 47.633°N, 59.256°W, pitfall trap, 25.VIII.2012, Lorna Lafosse (MUN) 9 females; same data except: 10.IX.2012 (MUN) 9 females; Salmon River near Main River, 51.174°N, 56.0181°W, tidal flats, under rocks/debris, 3.VII.2012, D. Langor & G. Pohl (MUN) 1 female; x.s. TCH & Terra Nova River, detritus on sand, 48.638°N, 54.039°W, 18.VIII.2014, D. & M. Langor (MUN) 1 female.
Reference	Klimaszewski et al. 2015c

##### Bionomics.

In Newfoundland, adults were collected in pitfall traps in boreal mixedwood and conifer forests and from under seashore detritus. In New Brunswick, adults were found in sphagnum moss and litter in calcareous eastern white cedar fens, in a black spruce forest, and one individual was collected from moldy conifer duff at the base of a large pine in a mixed forest (Klimaszewski et al. 2015). Adults were found in April and May in New Brunswick, and June to August elsewhere.

##### Comments.

This species is probably more widely distributed in the boreal forest of Canada. Some specimens from Cheeseman Provincial Park are tentatively associated with this species because the antecostal suture of female sternite VIII was not straight like in typical forms but was strongly sinuate medially. These specimens were excluded from *Mocyta fungi* (Gravenhorst) because of the short elytra, about as long as the pronotum, while the elytra are longer than the pronotum in *Mocyta fungi*.

#### Stethusa spuriella

Taxon classificationAnimaliaColeopteraStaphylinidae

(Casey)

Figs 101–108

Atheta (Stethusa) spuriella Casey, 1910: 8. As Stethusa: Gusarov 2003b: 239; Brunke et al. 2012: 181.

##### Diagnosis.

**Figures 101–108.**
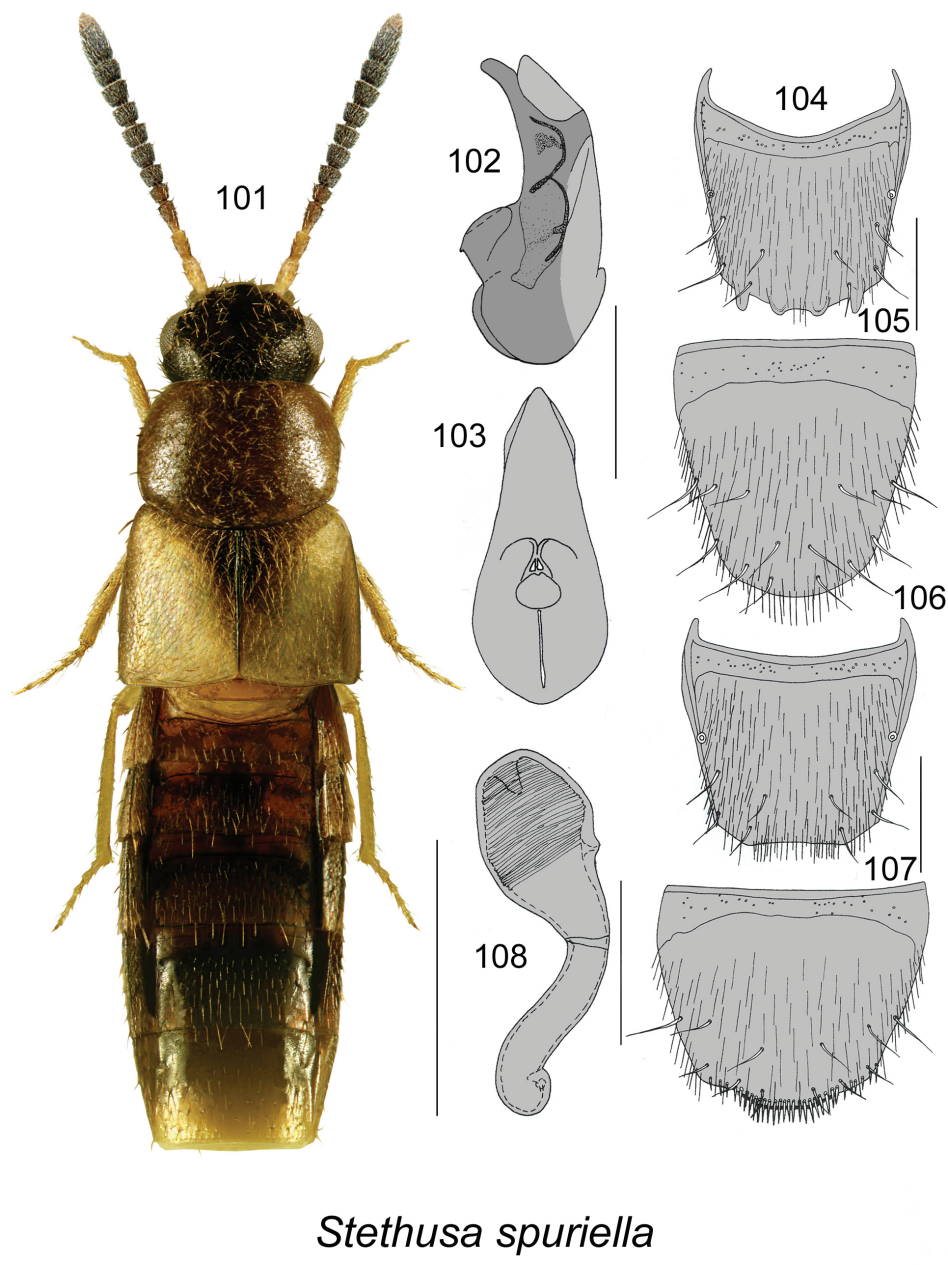
*Stethusa spuriella* (Casey): **101** habitus in dorsal view **102** median lobe of aedeagus in lateral view **103** median lobe of aedeagus in ventral view **104** male tergite VIII **105** male sternite VIII **106** female tergite VIII **107** female sternite VIII **108** spermatheca. Figures 102–108 after Gusarov (2003b). Scale bar of habitus = 1 mm; remaining scale bars = 0.2 mm.

This species may be distinguishable from two other Nearctic *Stethusa* species by the following combination of characters (Gusarov 2003b): *Stethusa spuriella* differs from *Stethusa dichroa* (Gravenhorst) in a smaller body size (length 2.1–2.5 mm), the lack of sub-basal impressions of the terminal antennal article (Fig. 101), the lack of the distal spines of the internal sac (Figs 102, 103); the shape of the spermatheca (Fig. 108); and the lack of a female accessory sclerite. *Stethusa spuriella* differs from *Stethusa klimschi* (Bernhauer) in having a smaller body size, the bent apex of the median lobe in lateral view (Fig. 102), and a shorter spermatheca (Fig. 108).

##### Distribution.

**Table:** 

Origin	Nearctic
Distribution	Canada: **NL**, ON; USA: DE, FL, GA, IN, MO, NY, OH, PA
New records	New provincial record; NEWFOUNDLAND: Barachois Pd. Prov. Pk., 48.483°N, 58.269°W, 11-VII-2011, mixed forest, pitfall trap, Heather Beck, (MUN) 1 female.
References	Casey 1910, Gusarov 2003b, Brunke et al. 2012

##### Bionomics.

In Newfoundland, one female was captured in a pitfall trap in mixed forest. In Ontario, *Stethusa spuriella* appears to be a common species in both forested and open habitats, some specimens were captured on fungi (Brunke et al. 2012). Adults were collected from May to August.

##### Comments.

This species probably reaches its northernmost distribution limit in Newfoundland.

#### Philhygra hygrotopora

Taxon classificationAnimaliaColeopteraStaphylinidae

(Kraatz)

Figs 109–115

Homalota hygrotopora Kraatz 1856: 220. As Philhygra: Palm 1970: 134; Webster et al. 2016.

##### Diagnosis.

**Figures 109–115.**
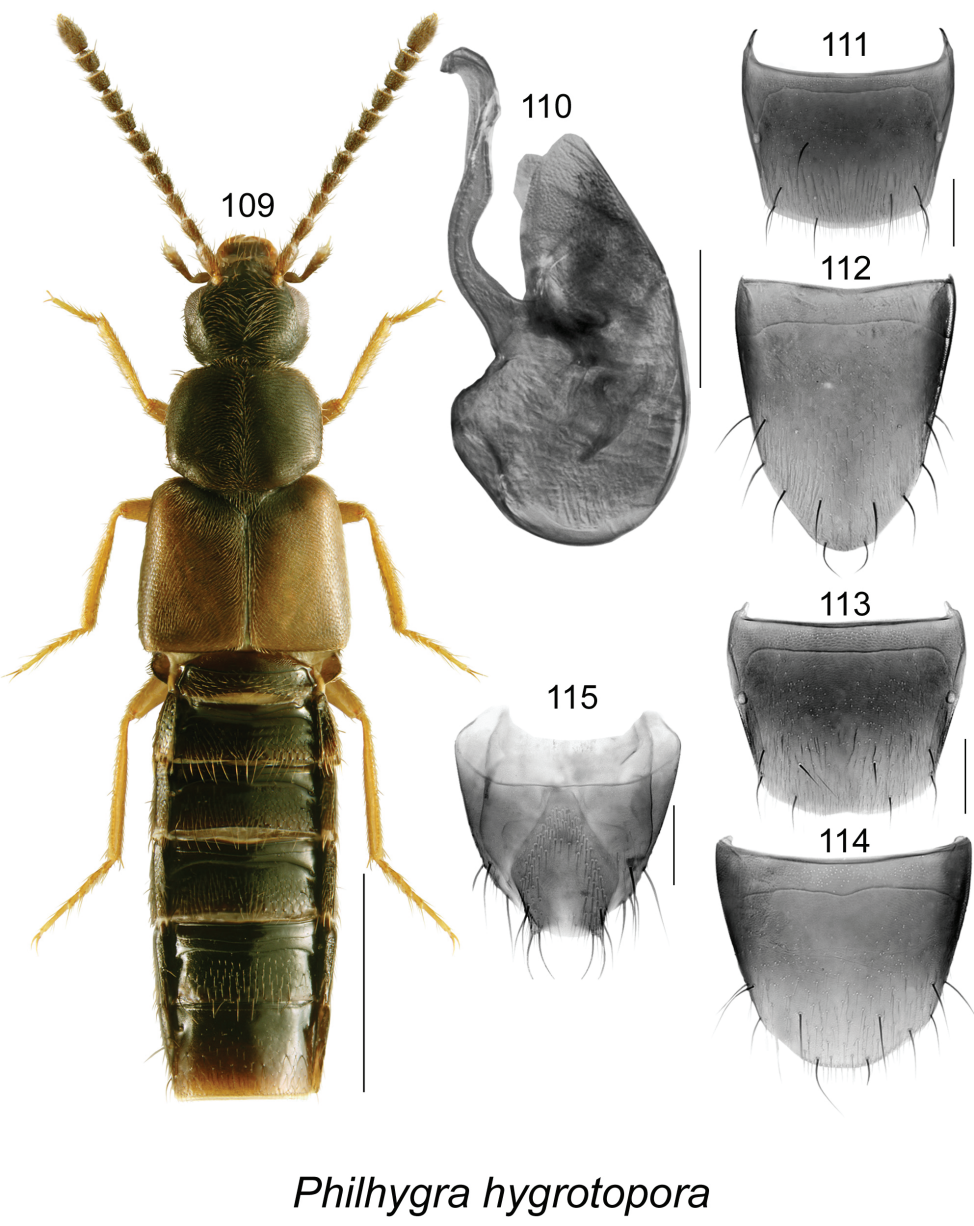
*Philhygra hygrotopora* (Kraatz): **109** habitus in dorsal view **110** median lobe of aedeagus in lateral view **111** male tergite VIII **112** male sternite VIII **113** female tergite VIII **114** female sternite VIII **115** female terminal segments (pygidium). Scale bar of habitus = 1 mm; remaining scale bars = 0.2 mm.

This species may be distinguishable from two other similar Nearctic species of *Philhygra* by the following combination of characters: body length 3.4 mm; body narrow with subparallel sides; antennae, head, pronotum, and abdomen dark brown, legs and elytra yellowish-brown (Fig. 109); integument not glossy; forebody with minute and dense punctation and dense pubescence (Fig. 109); head rounded postero-laterally, with large eyes; antenna with articles V-X slightly elongate to subquadrate (Fig. 109); pronotum rounded anteriorly and angular postero-laterally, transverse, slightly wider than head and slightly narrower than elytra, pubescence directed laterad on arcuate lines from midline of disc (Fig. 109); elytra slightly transverse, with pubescence directed postero-laterad and forming waves; abdomen subparallel, narrower than elytra. Median lobe of aedeagus and terminal abdominal structures as illustrated (Figs 110–115). For more details, see Webster et al. 2016.

##### Distribution.

**Table:** 

Origin	Palaearctic and adventive in Canada
Distribution	Canada: **NL**, NB
New records	New provincial record; NEWFOUNDLAND: St. John’s, Bowering Park, 47.525°N, 52.749°W, 30-VI-2010, in gravel/moss on riverbank, D. Langor & G.R. Pohl (LFC, MUN), 12 males, 4 females; same data as before, except: 1-VII-2010, in moss along riverbank, D. Langor (MUN) 1 male, 1 female; Searston, 47.828°N, 59.329°W, 23-VI-2010, under seaweed on sandy beach, D. Langor (MUN) 1 male; Newfoundland Drive, 47.6010°N, 52.7117°W, 20-VI-2009, 83 m, sweeping, D. Langor, (MUN) 1 female.
Reference	Webster et al. 2016

##### Bionomics.

In Newfoundland, specimens were found in gravel and moss on a riverbank, under seaweed on a sandy beach, and by sweeping vegetation in riparian habitat. In New Brunswick, *Philhygra hygrotopora* were found in moss near the splash zone of a waterfall, in gravel on the margin of a shaded spring-fed brook near a waterfall, among gravel on a gravel bar along a shaded brook in a northern hardwood forest, and in gravel along a cold shaded brook. A few individuals were found under decaying seaweed on a sea beach. Adults were collected during June, July, August, and September.

### HOMALOTINI Heer

#### Silusa prettyae

Taxon classificationAnimaliaColeopteraStaphylinidae

Klimaszewski & Langor sp. n.

http://zoobank.org/A558C4FA-F12B-4FEE-819B-71F69A5EEEF7

Figs 116–123

##### Holotype

(female). **Canada, Newfoundland**, Butterpot Provincial Park, 47.381°N, 53.044°W, pitfall trap, 26.IX.2012, Andrea Pretty (LFC).

##### Paratypes.

**Canada, Newfoundland**: Butterpot Provincial Park, 47.381°N, 53.044°W, pitfall trap, 11.VIII.2012, Andrea Pretty (LFC, MUN) 2 males, 1 female; same data except: 4.VIII.2012 (MUN) 1 female, 6.IX.2012 (MUN) 1 female.

##### Etymology.

This species is named after Andrea Pretty, an enthusiastic entomophilic park interpreter who collected the type series in Butterpot Provincial Park.

##### Diagnosis.

**Figures 116–123.**
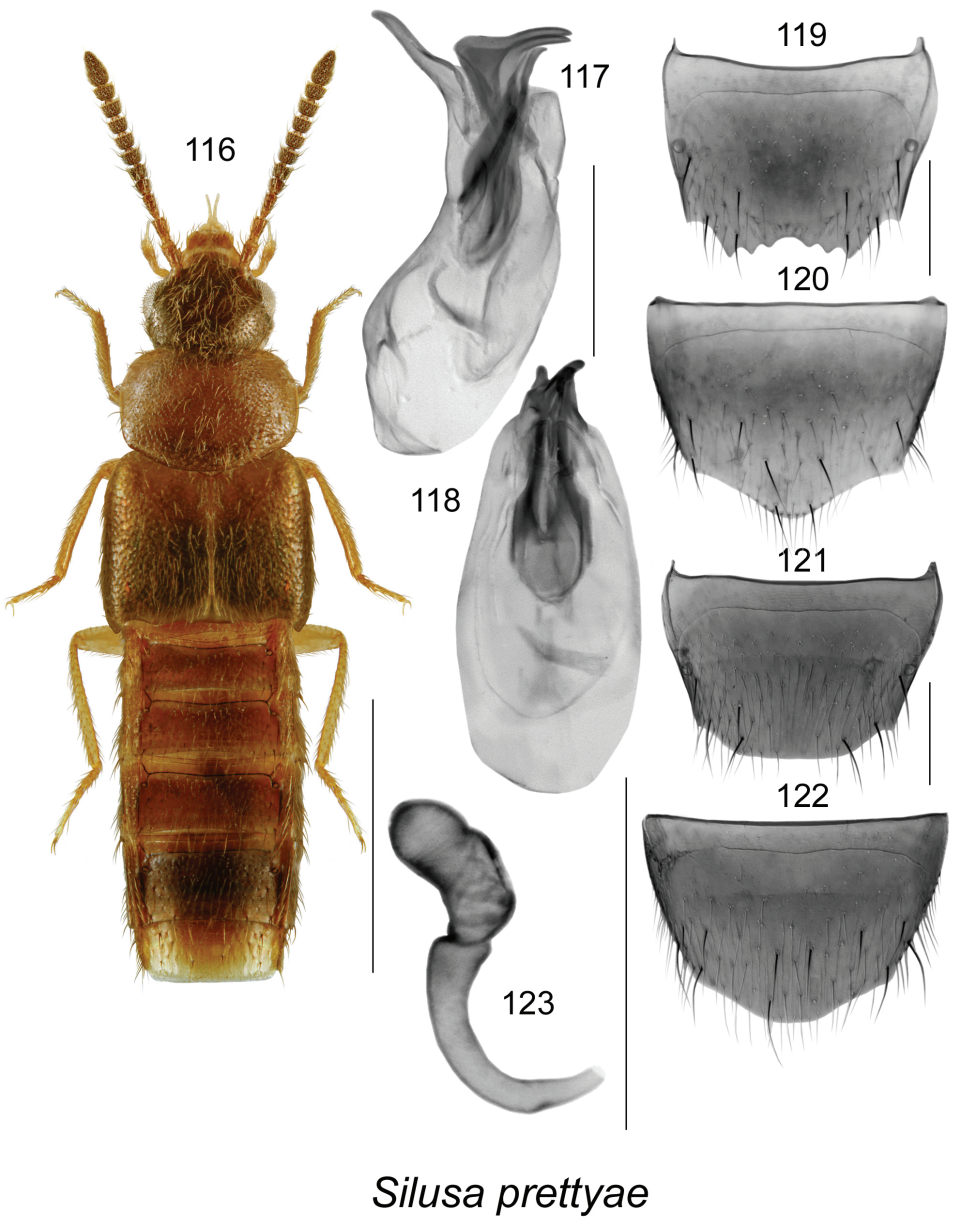
*Silusa prettyae* Klimaszewski & Langor, sp. n.: **116** habitus in dorsal view **117** median lobe of aedeagus in lateral view **118** median lobe of aedeagus in dorsal view **119** male tergite VIII **120** male sternite VIII **121** female tergite VIII **122** female sternite VIII **123** spermatheca. Scale bar of habitus = 1 mm; remaining scale bars = 0.2 mm.

Body length 2.7–3.0 mm; body moderately narrow, sides subparallel (Fig. 116); yellowish brown with head, antennae, posterior part of elytra and abdomen dark brown (Fig. 116); integument moderately glossy, sparsely punctate and pubescent, pubescence short and adhering to the body, forebody with dense meshed microsculpture, sculpticells hexagonal; head round, about as wide and as long as pronotum, with large eyes, as long as postocular area; antennae with articles I-III elongate and VI subquadrate, V-X transverse (Fig. 116); pronotum strongly transverse, slightly narrower at base than elytra; elytra longer than pronotum; abdomen broad, tapering apically. MALE. Median lobe of aedeagus with large oval bulbus and short, triangular tubus in dorsal view (Fig. 118); in lateral view, apical half of tubus produced ventrally at 75% angle (Fig. 117); two pairs of prominent internal sac structures (Figs 117–118); tergite VIII slightly emarginate apically and with broad teeth (Fig. 119); sternite VIII elongate, produced apically (Fig. 120). FEMALE. Tergite VIII truncate apically (Fig. 121); sternite VIII slightly produced apically (Fig. 122); spermatheca with tubular and apically spherical capsule without distinct apical invagination, stem arcuate, narrowed posteriorly (Fig. 123).

##### Distribution.

Known only from Butterpot Provincial Park in southeastern Newfoundland, Canada.

##### Bionomics.

Adults were collected in August and September in pitfall traps in coniferous boreal forest.

##### Comments.

This species is very similar externally to *Silusa californica* Bernhauer but may be separated from it by: its smaller body with shorter elytra (elytra at suture about as long as pronotum along median line); light brown colour with darker antennae, head, and posterior elytra and abdomen; and differently shaped spermatheca in lateral view (Fig. 123). The male of this species is similar to that of *Silusa californica* Bernhauer but the apical half of the tubus of the aedeagus is produced ventrally at about 75% angle and in *Silusa californica* at about 90% angle. The female spermatheca is distinct in its shape and has the best diagnostic features for this species (Fig. 123), and this is also the reason why the female was designated for a holotype. For illustrations of *Silusa californica*, see Klimaszewski et al. (2003). The three European species, *Silusa rubiginosa* (Er.), *Silusa rubra* (Er.), and *Silusa pipitzi* Epph., are ruled out as conspecific with *Silusa prettyae* as all three species have different proportions of forebody, and different body colour. For details see Lohse (1974).

### MYLLAENINI Ganglbauer

#### Myllaena procidua

Taxon classificationAnimaliaColeopteraStaphylinidae

Casey

Figs 124–131

##### Diagnosis.

**Figures 124–131.**
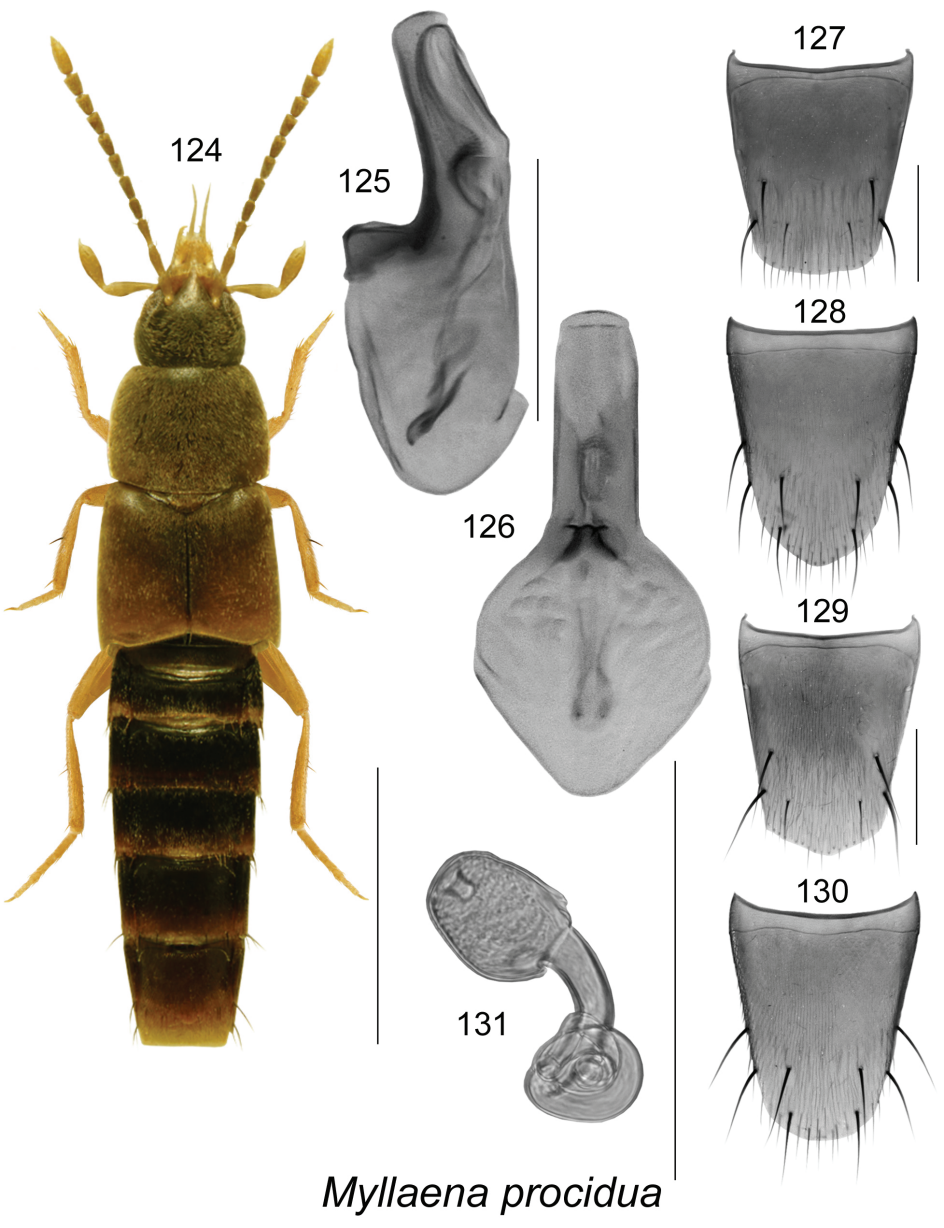
*Myllaena procidua* Casey: **124** habitus in dorsal view **125** median lobe of aedeagus in lateral view **126** median lobe of aedeagus in dorsal view **127** male tergite VIII **128** male sternite VIII **129** female tergite VIII **130** female sternite VIII **131** spermatheca. Scale bar of habitus = 1 mm; remaining scale bars = 0.2 mm.

This species may be distinguished by its body shape (Fig. 124), small size (about 1.6–2.3 mm long), antennal articles VII–X elongate (Fig. 124), and the shape of the median lobe of the aedeagus and the spermatheca (Figs 125, 126, 131). It is worthy to note that the median lobe of *Myllaena procidua* is similar to that of *Myllaena kaskaskia* Klimaszewski and *Myllaena vulpina* Bernhauer, but the shape of the spermatheca differs significantly and has much better diagnostic features for identification of this species.

##### Distribution.

**Table:** 

Origin	Nearctic
Distribution	Canada: **NL**, NB, QC. USA: MA, MD, VA
New records	New provincial record; **Canada**, NEWFOUNDLAND: Port au Port, Pen., Mainland, 48.5589°N, 59.1874°W, 9 m, 28-VII-2011, margin of stream, D. Langor & G. Pohl (MUN) 1 male; Blow Me Down, 49.049°N, 58.253°W, 400 m, banks of river, 26-VI-2010, D. Langor (MUN) 1 male.
References	Casey 1911, Klimaszewski 1982, Webster et al. 2009

##### Bionomics.

The Newfoundland specimens were taken on the gravel banks of a stream and a river. In New Brunswick, adults occurred along river (clear water) margins among cobblestones set in sand and fine gravel at the water’s edge, or among gravel at the edge of the water (Webster et al. 2009). Adults were located by turning over cobblestones and larger pebbles (Webster et al. 2009). In New Brunswick, adults were collected during May, June, July and August, by turning over cobblestones and pebbles (Webster et al. 2009).

## Supplementary Material

XML Treatment for Aleochara (Xenochara) inexpectata

XML Treatment for Aleochara (Aleochara) tahoensis

XML Treatment for Aleochara (Aleochara) gracilicornis

XML Treatment for Ilyobates bennetti

XML Treatment for Parocyusa americana

XML Treatment for Alevonota gracilenta

XML Treatment for Atheta (Dimetrota) giguereae

XML Treatment for Atheta (Pseudota) klagesi

XML Treatment for Atheta (Pseudota) pseudoklagesi

XML Treatment for Atheta (Thinobaena) pseudovestita

XML Treatment for Callicerus rigidicornis

XML Treatment for Mocyta luteola

XML Treatment for Mocyta sphagnorum

XML Treatment for Stethusa spuriella

XML Treatment for Philhygra hygrotopora

XML Treatment for Silusa prettyae

XML Treatment for Myllaena procidua
